# Cell division- and DNA replication-free reprogramming of somatic nuclei for embryonic transcription

**DOI:** 10.1016/j.isci.2021.103290

**Published:** 2021-11-03

**Authors:** Junko Tomikawa, Christopher A. Penfold, Takuma Kamiya, Risa Hibino, Ayumi Kosaka, Masayuki Anzai, Kazuya Matsumoto, Kei Miyamoto

**Affiliations:** 1 Laboratory of Molecular Developmental Biology, Faculty of Biology-Oriented Science and Technology, Kindai University, Wakayama 649-6493, Japan; 2 Department of Physiology, Development and Neuroscience, University of Cambridge, Downing Site, Cambridge CB2 3EG, UK; 3 Centre for Trophoblast Research, University of Cambridge, Downing Site, Cambridge CB2 3EG, UK; 4 Wellcome Trust – Medical Research Council Stem Cell Institute, University of Cambridge, Jeffrey Cheah Biomedical Centre, Puddicombe Way, Cambridge CB2 0AW, UK; 5 Institute of Advanced Technology, Kindai University, Wakayama 642-0017, Japan

**Keywords:** Cell biology, Stem cells research, Developmental biology

## Abstract

Nuclear transfer systems represent the efficient means to reprogram a cell and in theory provide a basis for investigating the development of endangered species. However, conventional nuclear transfer using oocytes of laboratory animals does not allow reprogramming of cross-species nuclei owing to defects in cell divisions and activation of embryonic genes. Here, we show that somatic nuclei transferred into mouse four-cell embryos arrested at the G2/M phase undergo reprogramming toward the embryonic state. Remarkably, genome-wide transcriptional reprogramming is induced within a day, and ZFP281 is important for this replication-free reprogramming. This system further enables transcriptional reprogramming of cells from *Oryx dammah*, now extinct in the wild. Thus, our findings indicate that arrested mouse embryos are competent to induce intra- and cross-species reprogramming. The direct induction of embryonic transcripts from diverse genomes paves a unique approach for identifying mechanisms of transcriptional reprogramming and genome activation from a diverse range of species.

## Highlights

• Mouse embryos arrested at the four-cell stage can induce transcriptional reprogramming
• ZFP281 plays a role in transcriptional reprogramming
• Embryonic environment permits cell division- and DNA replication-free reprogramming
• Transcriptional reprogramming of cross-species nuclei is induced in mouse embryos

## Graphical abstract

**Figure:**
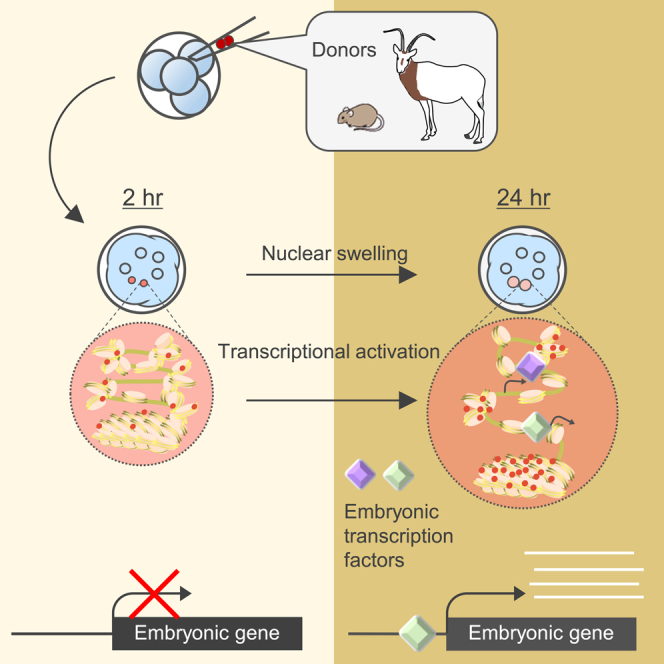

## Introduction

A major paradigm shift in our understanding of cell states began more than 50 years ago when Gurdon demonstrated that a differentiated cell state can be returned to an undifferentiated one by nuclear transfer (NT) of a somatic cell nucleus into an unfertilized egg (Gurdon, 1962). Later, this NT technique enabled cloning of various animal species, consolidating the idea that somatic cell nuclei can be reprogrammed in eggs or oocytes (Cibelli et al., 1998; Wakayama et al., 1998; Wilmut et al., 1997). Nuclear transfer, in theory, offers a unique opportunity to reprogram genomes of endangered animals for conservation (Loi et al., 2016). Reprogramming technologies have been further developed since the successful derivation of induced pluripotent stem cells (iPSCs) (Takahashi and Yamanaka, 2006) and are widely recognized as promising for regenerative medicine applications (Mandai et al., 2017). Although many of the factors and molecular mechanisms that induce somatic cell nuclear reprogramming have been studied using iPSCs (Cheloufi et al., 2015; Mikkelsen et al., 2008; Soufi et al., 2012), NT of somatic cells into oocytes remains the fastest and most efficient way to reprogram cells (Kim et al., 2010), indicating the potent reprogramming abilities of oocytes. Indeed, iPSC production was accelerated by overexpressing several factors enriched in oocytes or early embryos (Gonzalez-Munoz et al., 2014; Maekawa et al., 2011).

The remarkable reprogramming activity of oocytes was originally studied using conventional NT to an oocyte at the metaphase II (MII) stage. In this NT setup, a somatic nucleus transferred to MII oocytes undergoes DNA replication and cell division before activating embryonic genes. When cell and oocyte are derived from different species, the resulting NT embryo normally arrests without proceeding to embryonic gene activation. Therefore, a cross-species NT system using a *Xenopus* oocyte nucleus—or germinal vesicle (GV)—has been developed to study mechanisms of transcriptional reprogramming in oocytes, in which transcriptomes of transplanted mammalian nuclei are reprogrammed toward those of oocytes in cell division- and DNA replication-free manners (Jullien et al., 2014; Miyamoto et al., 2013, 2018). Ultimately, this system does not induce the transcriptional programs of early embryos, and it is therefore desirable to develop a system that allows for direct reprogramming of nuclei toward early embryonic states.

Conventional NT systems also hampers the proper understanding of the contribution of cell division and DNA replication to transcriptional reprogramming. The effects of cell division and DNA replication on transcriptional reprogramming have been studied using cell fusion- or iPSC-mediated reprogramming systems. After fusion of somatic cells with embryonic stem (ES) cells, DNA synthesis is required for full activation of a pluripotency gene *Oct4* from somatic nuclei (Tsubouchi et al., 2013), an observation also supported in iPSC studies (Koche et al., 2011). In addition, the only supporting evidence for cell division- and DNA replication-free reprogramming has been obtained in the *Xenopus* cross-species NT, leading to the suggestion that cell division and DNA replication are required for transcriptional reprogramming in mammals (Koche et al., 2011; Tsubouchi et al., 2013; Wang et al., 2014). It is therefore important to ask if cell division- and DNA replication-free reprogramming can be achieved in mammals. The development of such reprogramming system would also be necessary to fully understand the nature of the superior reprogramming activities of mammalian eggs/oocytes or early embryos.

In the present study, we devised a unique NT system using mouse four-cell embryos to induce transcriptional reprogramming without the need for cell division and DNA replication. We therefore named this NT as Nuclear Transfer to Elicit embryonic Transcriptional Reprogramming (NT-ETR). We demonstrate that NT-ETR enables transcriptional reprogramming even from cell lines and cross-species cells that had never before been successfully reprogrammed by conventional NT. Furthermore, NT-ETR provides a conclusive opportunity to ask if cell division and DNA replication are needed for NT-mediated transcriptional reprogramming. Our results show that cell division and DNA replication are not prerequisite for transcriptional reprogramming in mammalian embryos, supporting the notion that early embryos continue to possess the potent reprogramming abilities seen in oocytes, which is partially explained by the presence of embryonic transcription factors (TFs) such as ZFP281. Finally, our direct reprogramming system enables the reactivation of silenced genes in order to obtain genomic information of various animals including endangered or extinct species such as *Oryx dammah*.

## Results

### Mouse four-cell embryos arrested at G2/M phase as recipients for NT-ETR

**Figure 1:**
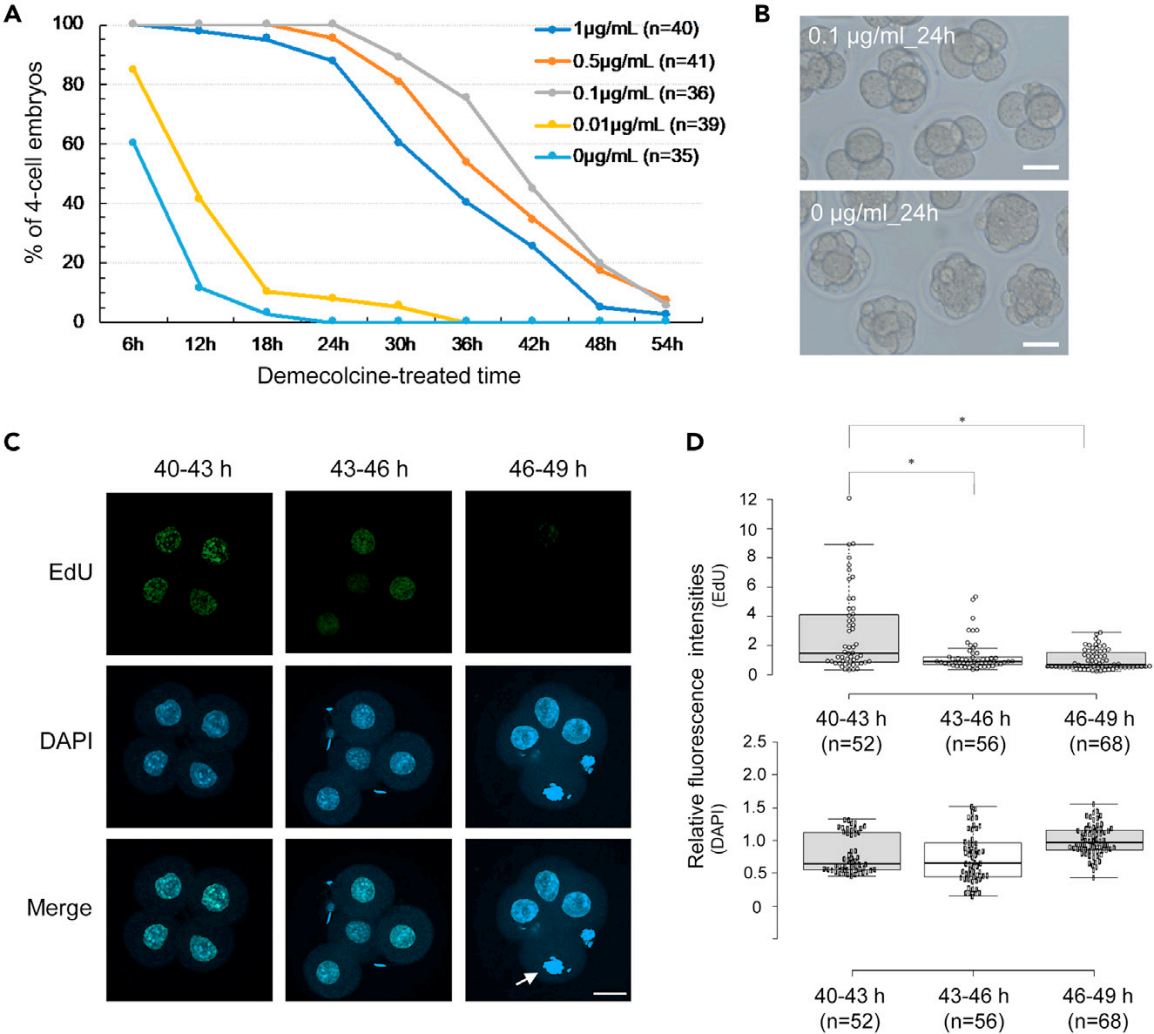
Conditions for inducing cell-cycle arrest at G2/M phase to mouse four-cell embryos (A) Percentages of four-cell-arrested embryos cultured with various concentrations of demecolcine. Four-cell embryos were treated with 0.01, 0.1, 0.5, or 1.0 μg/mL demecolcine from 40 hpi for 54 h, and four-cell embryos were morphologically assessed in each concentration group every 6 h. As a control, vehicle was added to the culture medium (0 μg/mL). Five independent experiments were performed. (B) Embryos treated with 0.1 μg/mL demecolcine or vehicle for 24 h from 40 hpi. Scale bars, 50 μm. (C) Assessment of DNA replication in demecolcine-treated four-cell embryos. DNA replication was observed by EdU incorporation during three different time frames; treatment with EdU from 40 to 43 hpi (40–43 h), 43 to 46 hpi (43–46 h), or 46 to 49 hpi (46–49 h). DNA was stained with DAPI. Three independent experiments were performed. Scale bar, 20 μm. (D) Quantitative assessment of EdU incorporation within four-cell embryonic nuclei. Fluorescence intensities of EdU and DAPI signals within each nucleus were determined as relative values to the mean nuclear fluorescence intensities for EdU and DAPI of 46–49 h. Tukey's HSD test was used to evaluate statistically significant differences (∗p < 0.05). Whiskers, 1.5x interquartile range; top box, 75^th^ percentile; center line, median; lower box, 25^th^ percentile.

We hypothesized that mouse embryos arrested at G2 phase, which occurs after DNA replication but prior to cell division, might possess reprogramming abilities and that mouse embryos at the four-cell stage could be used as recipients for NT since four-cell embryos retain a degree of totipotency (Boiani et al., 2019; Maemura et al., 2021) and begin to express pluripotency factors (Suzuki et al., 2015). To prepare the recipients for cell division- and replication-free NT, we first sought a condition that would enable arrest of four-cell embryos at G2 phase and identified treatment with demecolcine, which interferes with microtubule organization (Kapur et al., 2007), as a candidate. Four-cell embryos cultured in KSOM medium were treated with 0.01, 0.1, 0.5, or 1.0 μg/mL demecolcine from 40 h post insemination (hpi) for 54 h, and developmental progression was morphologically examined in each concentration group. Embryos treated with 0.1–1.0 μg/mL demecolcine stopped cell division at the four-cell stage, whereas 0–0.01 μg/mL demecolcine-treated embryos did not show developmental arrest (Figures 1A and 1B). In 0.5 and 1.0 μg/mL treatment groups, however, a certain number of embryos were degenerated after 24 h culture. From this quantitative time course assessment, we conclude that 0.1 μg/mL demecolcine was optimal for arrest of mouse four-cell embryos for at least 24 h without obvious morphological defects (Figure 1B).

We next determined the cell cycle states of four-cell embryos for subsequent NT experiments by assessing EdU incorporation. EdU was added to culture media during three different time frames; treatment with EdU from 40 to 43 hpi (40–43 h), 43 to 46 hpi (43–46 h), or 46 to 49 hpi (46–49 h). In the 40–43 h group, EdU incorporation was detected (Figure 1C). In the 43–46 h group, EdU signals were weak and significantly decreased compared with the 40–43 h group (Figures 1C and 1D, p < 0.05). In the 46–49 h group, almost no EdU signals were detected and blastomeres reaching M phase were observed (Figure 1C, arrow), indicating that DNA replication in four-cell embryos was complete before 46 hpi and hence embryos of 46–49 hpi are at G2/M phase. Taken together, mouse embryos can be arrested at the four-cell stage with 0.1 μg/mL demecolcine at least for 24 h and those with demecolcine are at G2/M phase from 46 hpi onward. In subsequent experiments, these conditions are used to prepare recipient embryos for achieving cell division- and replication-free reprogramming. Donor cell nuclei transplanted into the blastomeres of these arrested four-cell embryos can be incubated for a day, and such long exposure to the early embryonic environment might increase the chance of nuclear reprogramming.

### NT-ETR induces nuclear remodeling within a day

**Figure 2:**
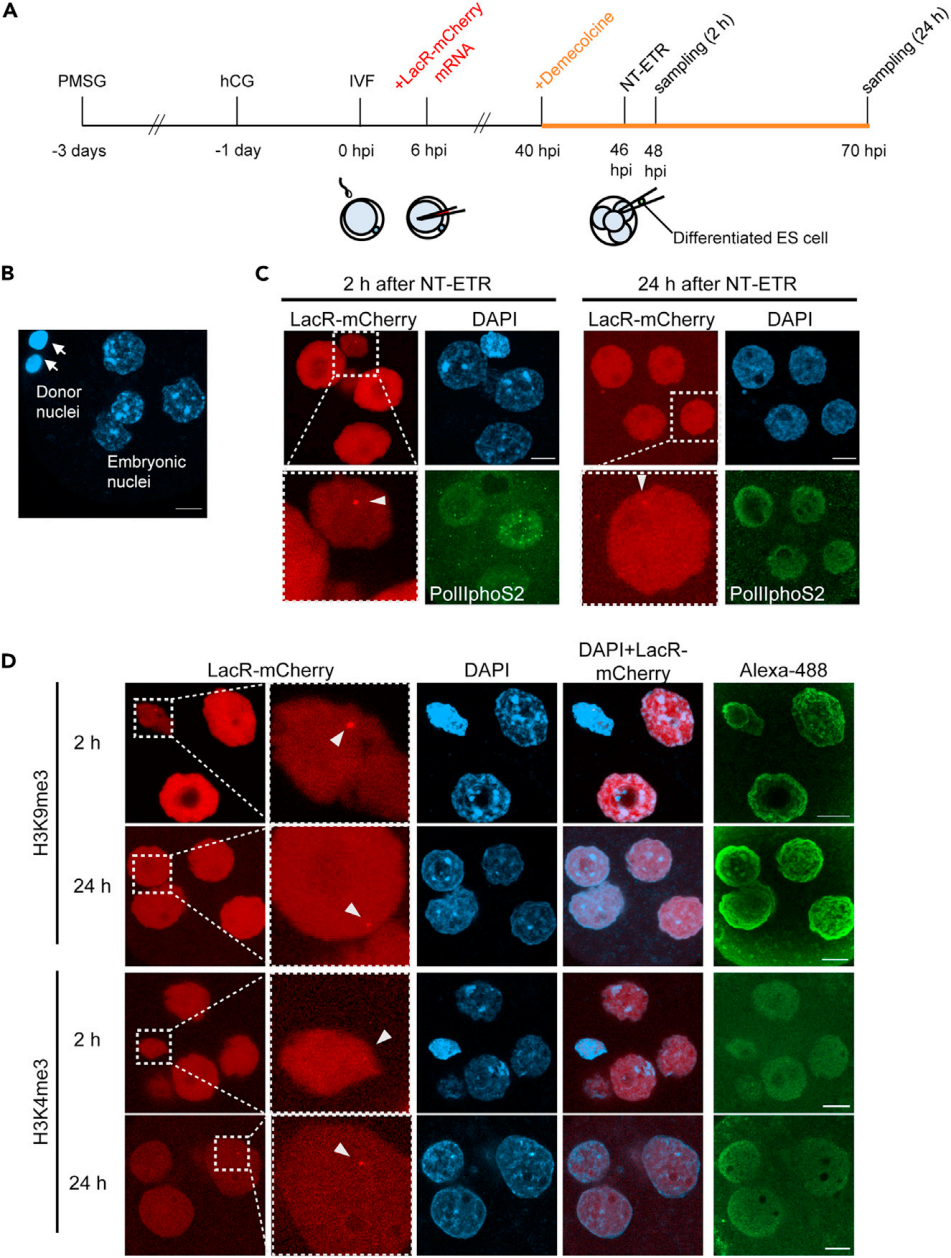
Remodeling of differentiated cell nuclei induced by NT-ETR (A) A schematic diagram of the time course of NT-ETR. *In vitro* fertilization (IVF)-derived mouse embryos were injected with Lac repressor mCherry (LacR-mCherry) mRNA solution at 6 hpi and cultured in KSOM medium containing 0.1 μg/mL demecolcine from 40 hpi. At 46 hpi, NT of differentiated NeoR5 ES cells was performed. The NT embryos were further cultured for 2 or 24 h and subjected to immunofluorescence staining. (B) Unfused donor cell nuclei (arrows) and recipient four-cell embryonic nuclei arrested at G2 phase were stained with DAPI. Scale bar, 10 μm. (C) Immunostaining of PolIIphoS2 (green) in the reconstructed embryos 2 and 24 h after NT-ETR. The NeoR5 donor cell nuclei were marked with accumulated LacR-mCherry (red dots; arrowheads). In the LacR-mCherry images, the bottom panels show higher magnification images of the regions indicated in the top panels by dashed boxes. Four independent experiments were performed. Scale bars, 10 μm. (D) Immunostaining of H3K9me3 and H3K4me3 (Alexa-488, green) in the reconstructed embryos 2 and 24 h after NT-ETR. In the LacR-mCherry images, the right panels show higher magnification images of the regions indicated in the left panels by dashed boxes. Arrowheads indicate the accumulated LacR-mCherry, marking the injected donor nuclei. Five independent experiments for H3K9me3 and six independent experiments for H3K4me3 were performed. Scale bars, 10 μm.

Differentiated donor cells were transferred to demecolcine-treated four-cell embryos and incubated for 24 h as depicted in Figure 2A. In order to trace nuclear remodeling of the transferred nuclei, differentiated ES cells carrying Lac repressor (LacR)-binding sites were used (Figure S1A, named as NeoR5 ES cell). This transgenic mouse ES cell line harbors 20–30 copies of Lac operator repeats, which can be visualized by the binding of LacR-mCherry (Miyamoto et al., 2011), and differentiation of the ES cells was induced by treatment with retinoic acid for 4 days. We first tested whether nuclear swelling, which is typically observed in reprogrammed nuclei after NT (Byrne et al., 2003), was induced in transferred nuclei. The nuclear sizes of four-cell embryos at G2 phase were much larger than those of donor cells, as revealed by DAPI staining of arrested four-cell embryos and unfused donor cells (Figure 2B, arrows). Two hours after NT-ETR, the donor cell nuclei, marked with accumulated LacR-mCherry, were slightly enlarged and decondensed (Figure 2C, arrowhead). The fused donor cell nuclei were further swelled at 24 h after NT-ETR and were indistinguishable from nuclei of recipient four-cell embryos (Figure 2C, arrowhead). We then monitored the process of nuclear swelling by live cell imaging. The injected NeoR5 cell showed accumulation of LacR-mCherry, as expected (Figure S1B, arrow). The injected nucleus was progressively enlarged during 500 min of incubation (Figure S1C and Video S1, arrows). This result suggests that nuclear swelling after NT-ETR proceeds gradually, in contrast to swelling after fertilization of sperm nuclei. Taken together, our results indicate that nuclear swelling, a landmark of nuclear reprogramming, is induced in differentiated nuclei after transplantation to four-cell embryos arrested at G2 phase.

Video S1. The swelling process of a differentiated cell nucleus injected into a four-cell embryo pre-loaded with mCherry-LacR, related to Figure 2 A position of an injected nucleus is indicated in Figure S1. Images were taken from 2 h after NT-ETR and every 10 min for 580 min. Scale bar, 20 μm2

We next asked if the arrested four-cell embryos and injected donor nuclei were transcriptionally active. The NT embryos were subjected to immunostaining with RNA polymerase II phosphorylated at Serine 2 (PolIIphoS2), elongating RNA polymerase II. Two hours after NT-ETR, PolIIphoS2 signals were clearly detected in embryonic nuclei at G2 phase but not in injected donor nuclei (Figure 2C, arrowhead), implying the detachment of somatic transcription machineries after NT (Jullien et al., 2014). Twenty-four hours after NT-ETR, the signals were observed in all nuclei of the NT embryos including the injected differentiated nucleus (Figure 2C, arrowhead). To summarize, four-cell embryos at G2 phase can induce nuclear remodeling and transcriptional activation to the transplanted somatic nuclei within a day.

### Epigenetic states of differentiated cell nuclei fused with four-cell embryos

To investigate whether nuclear remodeling of donor differentiated cells after fusing with four-cell embryos at G2 phase is accompanied by epigenetic changes, histone modifications in the transferred nuclei were examined by immunostaining. Trimethylation of histone H3 at lysine 9 (H3K9me3) is a typical mark of constitutive heterochromatin, whereas H3K4me3 is enriched in euchromatin. Two hours after NT-ETR, fused differentiated cell nuclei were still in the process of nuclear swelling and enriched with H3K9me3 predominantly at the inner nuclear membrane, unlike recipient embryonic nuclei in which broad H3K9me3 signals were detected (Figure 2D). Twenty-four hours later, H3K9me3 signals of injected nuclei were observed both at the nuclear periphery and inside the nucleus, resembling the pattern of embryonic nuclei. H3K4me3 signals were broadly detected in all nuclei including enlarged injected cell nuclei (Figure 2D, arrowheads show injected nuclei). These results suggest that nuclei of the fused differentiated cells are remodeled into similar epigenetic states of the embryonic ones without cell division and DNA replication at least on a global level.

### Transcriptional activation of arrested NT embryos

**Figure 3:**
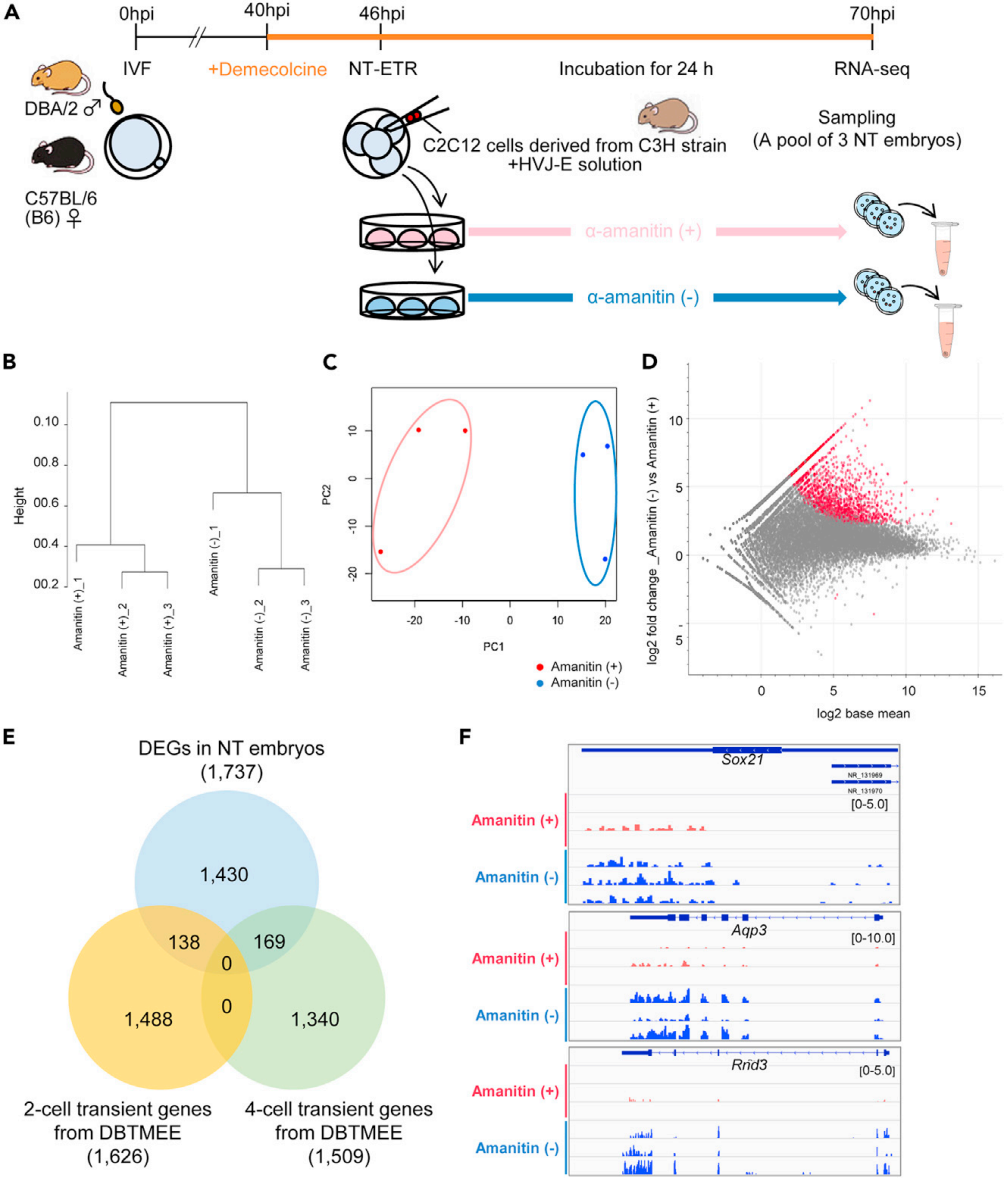
Activation of embryonic genes in the reconstructed embryos after NT-ETR (A) A schematic diagram of NT-ETR using three strains of mice. IVF-derived BDF1 mouse embryos were cultured in KSOM medium containing 0.1 μg/mL demecolcine from 40 hpi. At 46 hpi, NT of two C2C12 cells derived from C3H strain mouse was performed with HVJ-E solution. The NT embryos were further cultured for 24 h with or without α-amanitin and subjected to RNA-seq. Three independent biological replicates were performed. (B) Hierarchical clustering dendrogram generated using the gene expression profiles of NT embryos cultured with or without α-amanitin. (C) PCA of the gene expression profiles. Red and blue dots indicate the groups cultured with or without α-amanitin, respectively. (D) An MA plot on the gene expression levels. Red dots are the DEGs fulfilling two criteria: 4-fold change cutoff (α-amanitin (−) versus α-amanitin (+)) and the padj < 0.05. (E) A Venn diagram showing the numbers of total and overlapping genes among the DEGs, two- and four-cell transient genes. (F) Comparison of gene expression levels between α-amanitin-treated and untreated groups. *Sox21*, *Aqp3*, and *Rnd3* genes have been previously shown to be expressed in four-cell stage mouse embryos (Park et al., 2015).

Successful nuclear remodeling after NT-ETR prompted us to test whether genome-wide transcriptional reprogramming was induced after NT-ETR. Mouse C2C12 cells were fused with arrested four-cell embryos, and the NT embryos were treated with or without α-amanitin to block RNA polymerase II activities for detecting newly synthesized transcripts by Pol II (Figure 3A). After RNA-seq analyses of those embryos, we compared transcriptomes between each condition. Hierarchical clustering and principal component analysis (PCA) revealed that NT embryos with or without α-amanitin treatment showed a clearly different pattern of transcriptome (Figures 3B and 3C). An MA plot indicated that the vast majority of differentially expressed genes (DEGs) were identified as upregulated genes in NT embryos without α-amanitin treatment (Figure 3D and Table S1; 1,734 upregulated versus 3 downregulated genes). Gene ontology (GO) analyses revealed that genes related to transcription were upregulated (Figure S2A). We then compared DEGs with the Database of Transcriptome in Mouse Early Embryos (DBTMEE) (Park et al., 2015) and found that many genes transiently activated at the two- or four-cell stage, including *Sox21* and *Aqp3*, were upregulated (Figures 3E and 3F). A heatmap shows global upregulation of embryonically activated genes; 138 of two-cell and 169 of four-cell transient genes were upregulated in NT embryos (Figures S2B and S2C, respectively). Furthermore, Ingenuity Pathway Analysis (IPA) of DEGs revealed significantly changed canonical pathways and predicted upstream regulators. In canonical pathways, genes related to pluripotency features of ES cells were observed (Figure S2D). As predicted upstream regulators, TFs related to pluripotency and embryonic gene activation, including *Dux* (De Iaco et al., 2017), *Dppa4* (Eckersley-Maslin et al., 2019), and *Pou5f1* (known as *Oct4*), were identified (Table S2). Hierarchical clustering and PCA of repeat elements also showed clear differences between NT with and without α-amanitin treatment (Figures S2E and S2F). Repeat elements that are known to be specifically expressed in early embryos, such as MMSAT4 and MTA_Mm-int (Boroviak et al., 2018), were identified as newly expressed transcripts (Figure S2G; Table S3). These results indicate that embryonically activated genomic regions are actively transcribed in four-cell-arrested NT embryos. However, these analyses cannot distinguish transcripts derived from paternal, maternal, or somatic genomes, and therefore specific detection of transcripts from somatic genomes is required to conclude transcriptional reprogramming by NT-ETR.

### Transcriptional reprogramming of differentiated cells using NT-ETR without cell division and DNA replication

**Figure 4:**
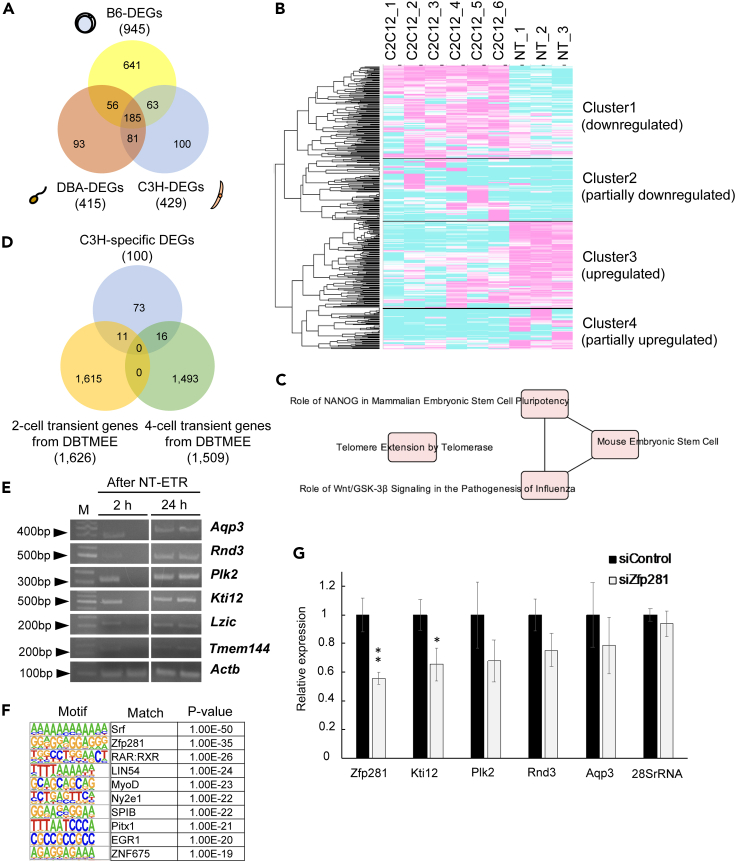
Transcriptional reprogramming of differentiated cells using NT-ETR (A) Venn diagram showing the numbers of total and overlapping genes identified as DEGs from B6, DBA/2, and C3H-derived genomes. (B) Heatmap shows the expression levels of C3H-DEGs in donor C2C12 cells and C2C12 cells after NT-ETR (429 genes in A). (C) Canonical pathways predicted by IPA using the cluster 3 genes in (B). (D) Venn diagram showing the numbers of total and overlapping genes among the C3H cell-specific DEGs, two- and four-cell transient genes. (E) RT-PCR analyses of candidate genes for transcriptional reprogramming marker 2 and 24 h after NT-ETR. M indicates DNA size marker (100-bp DNA ladder). Actb was used as an internal control. Three independent biological replicates were performed. (F) Enriched sequence motifs identified by the HOMER *de novo* motif analysis of the gene list that was activated only from C3H strain genomes after NT-ETR (100 DEGs shown in D). The top ten *de novo* motifs are shown. (G) qRT-PCR analyses of NT embryos in which Zfp281 was knocked down. Expression of transcriptional reprogramming markers was examined. Two independent experiments were performed. Data are represented as mean ± SEM. F- and T tests were performed. ∗p < 0.05, ∗∗p < 0.01.

To clarify the nature of transcriptional reprogramming we sought to identify the parental origin of transcribed DNA by taking advantage of unique genome sequences of inbred mice. In our NT-ETR system, three inbred strains of mice were used (Figure 3A): oocytes from C57BL/6 (B6), sperm from DBA/2, and donor C2C12 myoblasts from C3H strain mice. The numbers of B6, DBA/2, and C3H strain-derived newly transcribed genes after NT-ETR were 945, 415, and 429, respectively (Figure 4A; Tables S4, S5, and S6); among them, the number of strain-specific transcripts was 641, 93, and 100, respectively (Figure 4A). The upstream regulators identified by IPA from B6-specific transcripts included *Dux*, *Dppa4*, and *Zscan4c* (Table S7), whereas DBA/2 and C3H donor cell-specific transcripts led to the identification of *Pax7*, a male germline stem cell marker (Aloisio et al., 2014), and *MyoG*, a muscle-specific TF involved in the myogenesis (Tables S8 and S9). These transcription factors are candidate regulators that have been reported to activate/repress our detected genes, and the identification of *Pax7* and *MyoG* by this analysis implies that chromatin features of each genome affect transcriptional reprogramming. Of interest, however, GO analyses on each gene set revealed that embryonically expressed genes were significantly enriched in C3H donor cell-specific genes (100 genes) (Figure S3A). Indeed, genes that were silent in donor C2C12 cells were transcribed from the injected C2C12 nuclei (Figure 4B, clusters 3 and 4). Upregulated genes after NT-ETR compared with C2C12 transcriptome (cluster 3) were enriched with pluripotency and telomere elongation (Figure 4C), whereas senescence-related genes were downregulated after NT-ETR (Figure S3B). Furthermore, genes transiently activated at the two- or four-cell stage were transcribed from the C3H genome (Figures S3C–S3F). These results indicate that transcriptional reprogramming was indeed elicited in injected somatic cells by NT-ETR without cell division and DNA replication.

DEGs found only from C3H contained two- or four-cell transient genes (11 or 16 genes, respectively, Figure 4D). These genes were not identified as DEGs when B6 and DBA2 genomes were analyzed. We therefore hypothesized that these genes could be used as markers for transcriptional reprogramming of injected somatic nuclei. Among the C3H-derived two- and four-cell stage transient genes (27 genes), we selected 6 genes (*Aqp3, Rnd3, Plk2, Ktil2, Lzic*, and *Tmem144*) expressed at relatively high levels as the transcriptional reprogramming marker candidates. RT-PCR analyses using NT embryos revealed that expression of the majority of candidate genes increased at 24 h versus 2 h after NT-ETR (Figure 4E). Among them, expression of *Aqp3*, *Rnd3*, *Plk2*, and *Kti12* was abundantly detected at 24 h after NT-ETR in all NT embryos examined (Figure 4E). Together, these four genes may be used as transcriptional reprogramming markers for NT-ETR.

### Embryonic TFs responsible for transcriptional reprogramming

We next searched for TFs involved in transcriptional reprogramming after NT-ETR. We identified genes activated from each strain of genomes after NT-ETR (Figure 4A). Motif analyses were performed to find candidate TFs possibly bound to gene regulatory regions of those genes. ZFP281 and RAR binding motifs were found only in C3H-specific DEGs (Figures 4F and S4A). RAR has been shown to affect transcriptional reprogramming, possibly through opening of inaccessible sites in somatic chromatin such as *Pou5f1* and *Utf1* loci (Miyamoto et al., 2018), and RA activity in early embryos is required for embryonic gene activation (Iturbide et al., 2021). ZFP281, a zinc finger TF, maintains the pluripotency network in ES cells through the direct activation of *Nanog* and other pluripotency-related genes by binding to promoters with OCT4 and SOX2 (Kim et al., 2008; Wang et al., 2008). In our analysis, multiple ZFP281-binding sites were observed in two- and four-cell transient gene promoters such as *Lzic, Kctd9, Tmem144, Rnd3, Nck2*, and *Aqp3*. We then performed knockdown experiments of *Zfp281* in four-cell NT embryos by siRNA injection. Quantitative RT-PCR (qRT-PCR) analyses revealed that *Zfp281* transcripts were significantly reduced in the embryos injected with *Zfp281* siRNA compared with those with control siRNA. Expression levels of most reprogramming markers were also reduced (Figure 4G, 0.65-fold to 0.78-fold), among which *Kti12* gene expression was most affected (p < 0.05). In good agreement with these expression data, the *Kti12* gene locus had multiple consensus binding sites for ZFP281 within 10 kb upstream of its transcription start site (TSS) (Figure S4B), at which enhancer markers such as H3K4me1 and H3K27ac were enriched in mouse ES cells (Spicuglia and Vanhille, 2012). Thus, ZFP281 plays an important role in reactivating embryonic genes.

### DNA replication is not required for transcriptional reprogramming after NT-ETR using four-cell embryos

**Figure 5:**
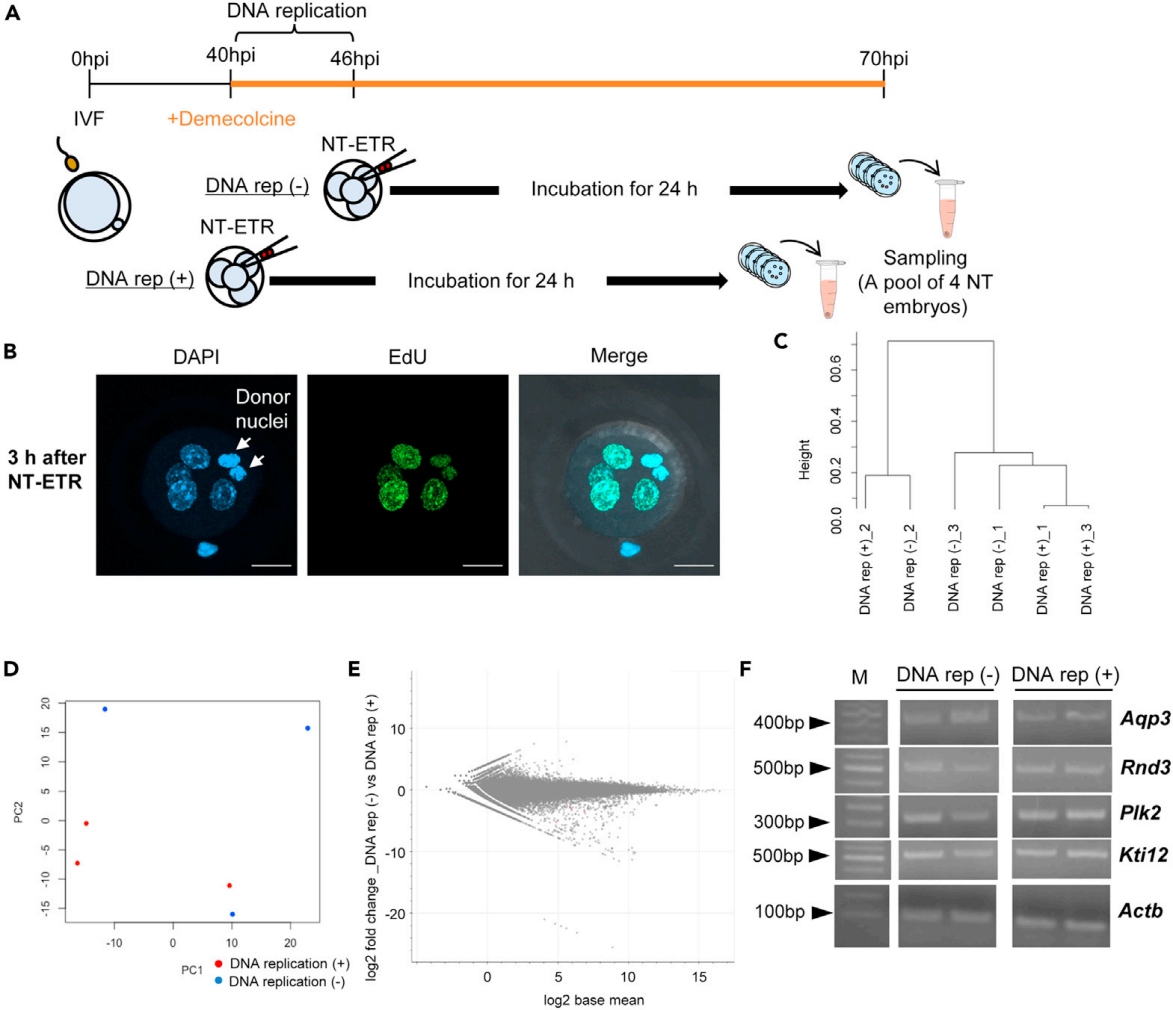
DNA replication prior to NT-ETR does not affect global transcriptional reprogramming (A) A schematic diagram of NT-ETR with or without DNA replication. At 40 or 46 hpi, NT of two C2C12 cells was performed with HVJ-E solution. Both reconstructed embryos were further cultured for 24 h and subjected to RNA-seq. Three independent biological replicates were performed. (B) EdU incorporation in the NT embryo in which two donor nuclei (arrows) were injected at 40 hpi. After culturing for 3 h from 40 hpi, embryos were subjected to EdU incorporation analysis to assess DNA replication. A representative image is shown (n = 19). Scale bars, 20 μm. (C) Hierarchical clustering dendrogram generated using the gene expression profiles of NT embryos with or without DNA replication. (D) PCA of the gene expression profiles. Red and blue dots indicate the groups with or without DNA replication, respectively. (E) An MA plot on the gene expression levels. Red dots are the DEGs. (F) RT-PCR analyses of transcriptional reprogramming marker genes 24 h after NT-ETR with or without DNA replication. Left gel images and DNA ladder markers (M) are the same as the ones used in Figure 4E (24 h). A representative gel image of three independent experiments is shown.

Our NT system used mouse four-cell embryos as recipient cells whose DNA replication was already complete before NT (Figures 1C and 1D). To investigate whether transcriptional reprogramming of donor differentiated cells elicited by fusing with four-cell embryos is susceptible to DNA replication, we performed NT-ETR before and after the period of DNA replication (Figure 5A). As described above, DNA replication of mouse four-cell embryos was accomplished before 46 hpi, so we performed NT at either 40 or 46 hpi; the former underwent DNA replication, whereas the latter did not (Figures 1D and 5B). RNA-seq analyses of these reconstructed embryos revealed that two groups (DNA rep (+) and DNA rep (−)) were not clearly distinguishable (Figures 5C and 5D). An MA plot also suggested that almost no significant difference in gene expression levels (1 upregulated and 4 downregulated genes) was observed between the presence and absence of DNA replication (Figure 5E). When the derivation of transcripts was separated to three strains (B6, DBA/2, and C3H), no significant differences (q < 0.05) were detected in expression of the genes from all strains of genomes. The transcriptional reprogramming markers, *Aqp3*, *Rnd3*, *Plk2*, and *Kti12*, also showed no differences in their expression levels (Figure 5F), indicating that transcriptional reprogramming of C3H donor cell nuclei was elicited regardless of the presence or absence of DNA replication. These results suggested that DNA replication is not necessarily required for the transcriptional reprogramming in four-cell embryos.

### Nuclear transfer to two-cell embryos induces activation of zygotic genome activation genes

**Figure 6:**
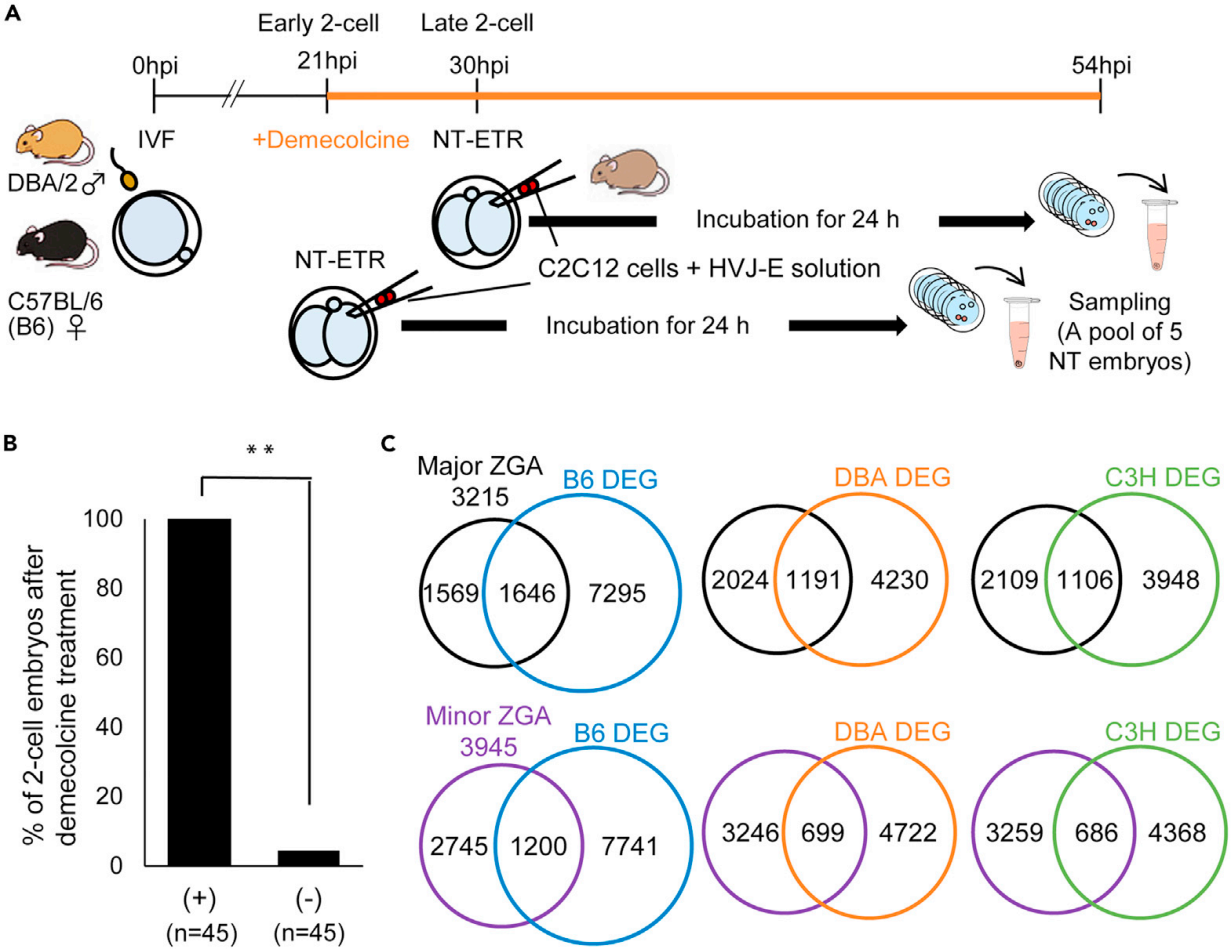
Activation of ZGA genes after NT to early two-cell embryos (A) A schematic diagram of NT-ETR using two-cell embryos. The NT embryos were further cultured for 24 h with or without α-amanitin and subjected to RNA-seq. Three independent biological replicates were performed. (B) Two-cell embryos were arrested at G2/M phase with demecolcine for 24 h. Three independent biological replicates were performed. Fisher’s exact test was performed. ∗∗p < 0.01. (C) Venn diagrams showing the numbers of total and overlapping genes between the DEGs identified in B6, DBA/2, and C3H-derived genomes and genes activated at major and minor ZGA, which are extracted from DBTMEE (Park et al., 2015).

Following successful reprogramming using four-cell embryos, we asked if other preimplantation stages of embryogenesis also possessed reprogramming activities using a similar experimental setup. Two-cell embryos were used as recipients for NT-ETR since major zygotic genome activation (ZGA) is induced at the two-cell stage. Treatment with 0.1 μg/mL demecolcine from 21 hpi onward successfully arrested two-cell embryos (Figures 6A and 6B). After NT-ETR with the arrested two-cell embryos at G2/M phase, transcriptional activation was examined by RNA-seq by comparing α-amanitin-treated and non-treated embryos. Unexpectedly, only a limited number of genes were activated from oocyte, sperm, and donor cell genomes when NT-ETR was performed at 30 hpi using late two-cell embryos; 46, 12, and 8 genes were activated, respectively. We then fused C2C12 cells with early two-cell embryos at 21 hpi, prior to DNA replication (Kang et al., 2014), and investigated gene activation. Of interest, a larger number of genes were transcribed and more than a thousand major ZGA genes were newly transcribed from C3H donor genomes (Figure 6C). These results suggest that late two-cell embryos are not capable of inducing genome-wide reprogramming, whereas transfer of somatic cells to early two-cell embryos allows transcriptional reprogramming. Four-cell embryos might therefore represent more efficient sources for the induction of replication-free transcriptional reprogramming.

### Transcriptional reprogramming of cells from *Oryx dammah* by NT-ETR

**Figure 7:**
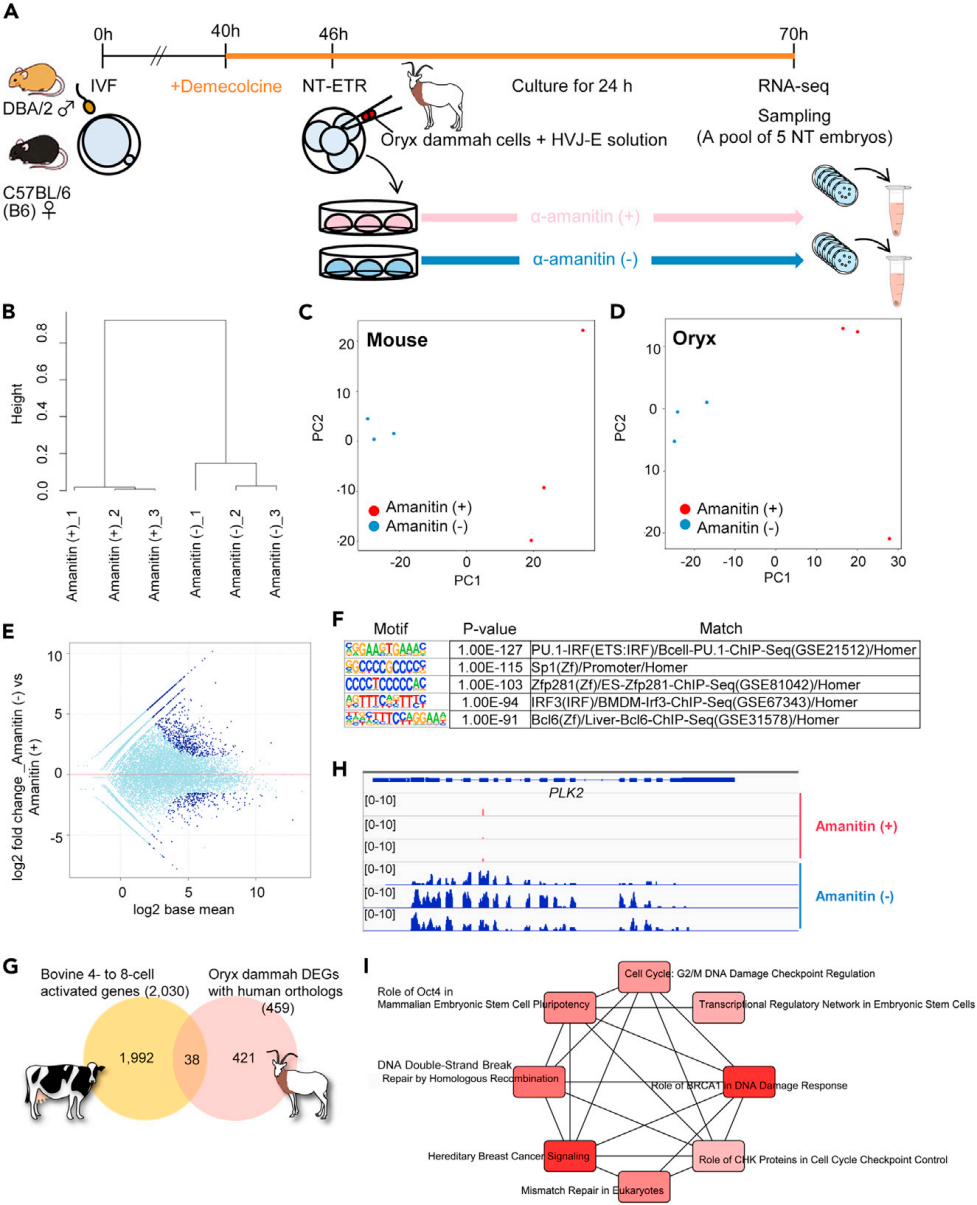
Activation of *Oryx dammah* genes in the reconstructed embryos after NT-ETR (A) Schematic diagram of NT-ETR using *Oryx dammah* cells. The NT embryos were further cultured for 24 h with or without α-amanitin and subjected to RNA-seq. Three independent biological replicates were performed. (B) Hierarchical clustering dendrogram generated using the gene expression profiles of NT embryos cultured with or without α-amanitin. (C and D) PCA of the gene expression profiles from mouse and *Oryx dammah* transcripts. Red and blue dots indicate the groups cultured with or without α-amanitin, respectively. (E) An MA plot on the gene expression levels of *Oryx dammah*. Blue dots are the DEGs. (F) Enriched sequence motifs from the gene list (883 of *Oryx dammah* DEGs shown in Figure 6E, blue dots). The top five motifs are shown. (G) Venn diagram showing the numbers of total and overlapping genes between the *Oryx dammah* DEGs and genes activated at four- to eight-cell stage in bovine embryos. (H) Comparison of *PLK2* gene expression levels between α-amanitin-treated and untreated groups after NT-ETR using *Oryx dammah* cells. (I) Canonical pathways predicted by IPA using the *Oryx dammah* orthologs corresponding to human genes (459 of *Oryx dammah* DEGs).

Since NT-ETR allowed cell division- and replication-free reprogramming, we sought to test if embryonic genes could be directly transcribed from cross-species cells of an endangered animal by bypassing the issue of developmental arrest of NT embryos. We collected cells from postmortem *Oryx dammah*, a species of oryx that is extinct in the wild. Bovidae to which *Oryx dammah* belongs has diverged from rodents approximately 100 million year ago (Jones et al., 2019). After NT-ETR of two *Oryx dammah* cells to mouse four-cell embryos, the fused *Oryx dammah* cell nuclei were swelled and were indistinguishable from nuclei of recipient mouse four-cell embryos 24 h after NT-ETR (Figure S5A, arrowheads indicate total six nuclei). Immunostaining with PolIIphoS2 revealed that all nuclei including *Oryx dammah* nuclei were transcriptionally active (Figure S5A). Next, RNA-seq analyses were performed as depicted in Figure 7A. Sequence reads were divided into those from mouse or *Oryx dammah*, and the transcriptome of each species was greatly altered by blocking PolII activities with α-amanitin (Figures 7B–7D), indicating that PolII-dependent transcription was induced after NT-ETR. We identified 883 DEGs from *Oryx dammah* transcripts (729 upregulated versus 154 downregulated genes, padj < 0.05) (Figure 7E, blue dots). TF motif analyses again detected ZFP281 using *Oryx dammah* DEGs (Figure 7F). We then searched for unique human orthologs against 883 DEGs, resulting in the 459 ortholog genes. This list matched 38 genes with the four- to eight-cell activated gene list of *Bos taurus* (Jiang et al., 2014), a closely related species of *Oryx dammah* (Figure 7G), and notably included *PLK2*, a reprograming marker found in mouse intraspecies NT (Figures 4E and 7H). Canonical pathway analysis by IPA revealed that genes related to pluripotency features of ES cells were enriched in *Oryx dammah* DEGs (Figure 7I). We further characterized *Oryx dammah* DEGs by analyzing their biological functions, and “Gene expression” term was enriched (Figures S5B and S5C), implying that genes related to transcriptional activation were activated in *Oryx dammah* genomes after NT-ETR. Furthermore, as the upstream regulator for *Oryx dammah* DEGs, let-7 miRNA and *POU5F1* were identified by IPA (Table S10), which are well-known regulators of embryonic pluripotency (Rahkonen et al., 2016), and *Oryx dammah* DEGs contained many target genes for let-7 (Figure S5D). These results demonstrate that NT-ETR allows transcriptional reprogramming of cross-species cells derived from an endangered animal.

## Discussion

Cell division- and DNA replication-free reprogramming has been exclusively studied using the interspecies nuclear transfer system of *Xenopus* oocytes. A number of reprogramming mechanisms and factors have been identified and, of note, many such identified factors also play crucial roles in other intraspecies reprogramming systems (Jullien et al., 2014; Miyamoto et al., 2011, 2018). These studies undoubtedly present the usefulness of a cell division- and DNA replication-free reprogramming system. However, an equivalent has never been developed in mammals, leading to the suggestion that cell division and DNA replication are prerequisite for transcriptional reprogramming in mammals using a different experimental setup (Koche et al., 2011; Tsubouchi et al., 2013; Wang et al., 2014). Here, we have developed cell division- and DNA replication-free NT-ETR system using mouse four-cell stage embryos, inducing transcription of embryonically activated genes from differentiated cells (Figure S6). This ability to induce embryonic transcription is an important difference from the *Xenopus* oocyte NT system, in which oocyte transcriptional programs are induced to the transplanted nuclei (Jullien et al., 2014). Remarkably, NT-ETR even achieved transcriptional reprogramming of cells that have never been reprogrammed by a conventional nuclear transfer method in mammals.

The cell division- and DNA replication-free nuclear transfer in *Xenopus* utilized oocytes arrested at the GV stage, known to be exceptionally active in transcription (Byrne et al., 2003). However, grown GV oocytes in mammals, whose chromatin surrounds nucleoli, are transcriptionally silent (Bouniol-Baly et al., 1999) and therefore a similar experimental setup to *Xenopus* is difficult to recapitulate. Furthermore, embryos become transcriptionally active only after zygotic genome activation (Vastenhouw et al., 2019), and the cell cycle rapidly progresses in preimplantation embryos without arrest. These features of mammalian oocytes and embryos hampered the development of the cell division- and DNA replication-free reprogramming system. We therefore artificially induced cell-cycle arrest of mouse embryos at G2 phase using demecolcine to let arrested embryos maintain transcriptional activities. This was achieved because early embryonic cell cycles progress synchronously. In our experiments, four-cell embryos, but not two-cell embryos, at G2 phase induced genome-wide transcriptional reprogramming of transplanted somatic genomes. When two-cell embryos were used as recipients, only after transfer to early two-cell embryos were many of the major ZGA genes activated (Figure 6). It is known that minor ZGA is induced during the late one-cell and the early two-cell stage and minor ZGA is required for the subsequent induction of major ZGA (Abe et al., 2018). Therefore, the failure to activate major ZGA genes after nuclear transplantation to the late two-cell embryos might be due to the lack of a preceding and prerequisite minor ZGA for donor genomes. Indeed, NT to early two-cell embryos allowed activation of minor ZGA genes (Figure 6C). It is speculated that minor ZGA globally relaxes chromatin structures and/or activates TFs responsible for subsequent major ZGA such as *Dux* (Eckersley-Maslin et al., 2018). Alternatively, late two-cell embryos might lack factors responsible for transcriptional reprogramming. Our NT system would be useful for dissecting mechanisms of ZGA.

One characteristic feature of cell division- and DNA replication-free reprogramming after NT-ETR is the remodeling of the injected differentiated nuclei. Donor cell nuclei injected into recipient embryos became synchronized in the cell cycle of the recipient embryos. The nuclei of donor cells fused with G2 phase-arrested embryos gradually swelled (Figure S1 and Video S1), remained at interphase, and finally expanded to the size of four-cell embryonic nuclei with epigenetic changes and transcriptional reprogramming within 24 h (Figures 2, 3, and 4). In a conventional nuclear transfer system, transplanted nuclei undergo a marked swelling through an influx and egress of proteins within the egg accompanied by cellular reprogramming (Fujita and Wade, 2004). Presumably, a similar scenario can be drawn in our NT-ETR. However, decondensing factors such as nucleoplasmin and H1foo should be more enriched in one-cell embryos than in four-cell embryos (Kunitomi et al., 2016; Tamada et al., 2006). One possible explanation for the gradual increase in the nuclear size is that nuclear swelling is mediated by a recently found player, nuclear actin (Baarlink et al., 2017; Wei et al., 2020). We have shown that pronuclear volume is reduced when nuclear actin is depolymerized (Okuno et al., 2020). Furthermore, in the cultured cells, nuclear swelling and chromatin decondensation after mitosis are mediated by nuclear actin assembly (Baarlink et al., 2017). Nuclear actin polymerization is also important for transcriptional reprogramming in *Xenopus* oocytes (Miyamoto et al., 2011). Thus, nuclear actin could directly contribute to not only gradual swelling of the transferred nuclei after NT but also the nuclear reprogramming.

To explore the factors necessary for the successful reprogramming of differentiated cells by NT-ETR, TF binding-site motifs enriched among the strain (C57BL/6, DBA/2, or C3H)-specific transcripts were identified (Figures 4D and S4). Of interest, different TFs seem to regulate transcription in different genomes. Of note, pluripotency-related protein Zfp281 (Kim et al., 2008; Wang et al., 2006, 2008) was detected only from donor cell-specific transcripts. Indeed, the knockdown of Zfp281 resulted in downregulation of two- and four-cell activated genes that contain Zfp281-binding sites at gene regulatory regions (Figures 4G and S4B). These results suggest that our NT-ETR system can be used to identify unique TFs and/or transcription networks that are associated with four-cell stage mouse embryos. Once NT-ETR is extended to other developmental stages such as morula embryos, this experimental setup can be a powerful tool to decipher developmental stage-specific transcriptional regulation.

Finally, NT-ETR was utilized to induce transcriptional reprogramming of cross-species cells of an endangered animal. It is extremely difficult or almost impossible to collect germ cells from endangered animals and therefore NT of somatic cells has been regarded as a promising approach to propagate genetic materials from those animals (Beyhan et al., 2007). However, reprogramming of such species has been challenging since available recipient oocytes from model animals were not able to reprogram cells of most wild animals using the conventional NT, except in rare cases where recipient oocytes of closely related species were available or bovine oocytes were used as recipients (Wang et al., 2009, 2011). Therefore, an alternative approach that allows reprogramming of cells derived from various species is desirable. We propose that our NT-ETR serves as an attractive choice for the purpose of studying and preserving functional information of genomes derived from endangered and even extinct species. We showed that mouse four-cell embryos are able to reprogram embryonic gene transcription from the *Oryx dammah* genome (Figure 7). These results imply that NT-ETR makes it possible to reprogram cells of distinct species. Gene regulatory functions of genomes are exerted even in the cross-species environment, as evident from the human-mouse hybrid cell experiments (Wilson et al., 2008). Since our presented NT-ETR allows xenogenic transcription, functional genomic information of various species can be obtained. This unique approach will pave the way for comparing genomic functions of wild and even extinct animals as long as genomic materials are available, complementing and further extending the genomic sequence information. Furthermore, mouse embryos can be cryo-preserved for the storage of valuable genomes, and future studies might even enable the propagation of the injected somatic nuclei to later stages, since demecolcine can work as a reversible inhibitor. In conclusion, the NT system developed here allows the direct induction of embryonic transcripts from intra- and interspecies nuclei and provides the proof of concept that transcriptional reprogramming can be achieved without the need for cell division and DNA replication in mammalian embryos.

### Limitations of the study

This study shows the NT-ETR method, which enables to induce transcription of embryonic genes from intra- and cross-species cells. Although the injected somatic nuclei are reprogrammed toward the early embryonic state without the need for cell division and DNA replication, we have not shown a method to re-initiate the development of the NT embryos for propagation of the injected nuclei. Since recipient four-cell embryos were reversibly arrested with demecolcine, future studies might enable the propagation of cross-species nuclei for preserving genomic materials. The developmental potential of the reprogrammed nuclei by NT-ETR has not been tested as well, and it would be also interesting to ask whether such nuclei support efficient development of cloned embryos.

## STAR★Methods

### Key resources table

**Table:** 

REAGENT or RESOURCE	SOURCE	IDENTIFIER
**Antibodies**		

Rabbit Anti-H3K4me3 Polyclonal antibody, Unconjugated (1:500 IF)	Active Motif	Active Motif Cat# 39159; RRID: AB_2615077
Rabbit Anti-H3K4me3 Polyclonal antibody, Unconjugated (1:500 IF)	Abcam	Abcam Cat# ab8580; RRID: AB_306649
Rabbit Anti-H3K9me3 Polyclonal antibody, Unconjugated (1:300 IF)	Abcam	Abcam Cat# ab8898; RRID: AB_306848
Rabbit Anti-H3S10ph Polyclonal antibody, Unconjugated (1:200 IF)	Abcam	Abcam Cat# ab5176; RRID:AB_304763
Mouse Anti-RNA Polymerase II H5 monoclonal antibody, Unconjugated (1:100 IF)	Covance	Covance Cat# MMS-129R; RRID:AB_10143905
Goat anti-Mouse IgM (Heavy chain) Cross-Adsorbed Secondary Antibody, Alexa Fluor 488 (1:2,000 IF)	Thermo Fisher Scientific	Thermo Fisher Scientific Cat# A-21042, RRID:AB_2535711
Goat anti-Rabbit IgG (H+L) Cross-Adsorbed Secondary Antibody, Alexa Fluor 488 (1:2,000 IF)	Thermo Fisher Scientific	Thermo Fisher Scientific Cat# A-11008; RRID:AB_143165

**Bacterial and virus strains**		

Hemagglutinating virus of Japan envelope (HVJ-E) solution	Ishihara Sangyo Kaisha	CF004

**Chemicals, peptides, and recombinant proteins**		

Demecolcine	SIGMA-Aldrich	D7385
α-amanitin	MERCK	A2263-1MG
VECTASHIELD Mounting Medium with DAPI antibody	Vector Laboratories	H-1200

**Critical commercial assays**		

PicoPure™ RNA Isolation Kit	Thermo Fisher Scientific	KIT0204
SuperScript™ III First-Strand Synthesis System	Thermo Fisher Scientific	18080051
Click-iT™ Plus EdU Cell Proliferation Kit for Imaging, Alexa Fluor™ 488 dye	Thermo Fisher Scientific	C10637
SMART-seq v4 Ultra Low Input RNA Kit	TaKaRa	24888N
Nextera DNA Sample Preparation Kit	Illumina	FC-131-1024
ERCC RNA Spike-In Mix	Thermo Fisher Scientific	4456740

**Deposited data**		

RNA-seq	This paper	GEO: GSE162345
C2C12 RNA-seq data	Picelli et al. (2013)	GEO: GSE49321
ES ChIP-seq data (H3K27ac)	ENCODE	GEO: GSM769009
ES ChIP-seq data (H3K4me3)	ENCODE	GEO: GSM1000099
C2C12 ChIP-seq data (H3ac)	ENCODE	GEO: GSM918422

**Experimental models: Cell lines**		

Mouse ES cell line; NeoR5	Miyamoto et al. (2011)	N/A
Mouse C2C12 cell line	Miyamoto et al. (2011)	N/A
*Oryx dammah* cell	This paper	N/A

**Experimental models: Organisms/strains**		

Mice C57BL/6 and DBA/2	CLEA Japan (Tokyo, Japan)	N/A

**Oligonucleotides**		

Primers for RT-PCR and qPCR	This paper	STAR Methods
LacR-mCherry mRNA	Miyamoto et al. (2011)	N/A

**Software and algorithms**		

ZEN	Carl Zeiss	https://www.zeiss.co.jp/microscopy/products/microscope-software/zen.html
Trimmomatic	Bolger et al. (2014)	http://www.usadellab.org/cms/?page=trimmomatic
trim_galore	Babraham institute	http://www.bioinformatics.babraham.ac.uk/projects/trim_galore/
cutadapt	Martin (2011)	https://cutadapt.readthedocs.io/en/stable/
STAR	Dobin et al. (2013)	https://github.com/alexdobin/STAR
DESeq2	Love et al. (2014)	https://bioconductor.org/packages/release/bioc/html/DESeq2.html
Kallisto software	Bray et al. (2016)	https://github.com/pachterlab/kallisto/releases
Sleuth	Pimentel et al. (2017)	https://github.com/pachterlab/sleuth
Database of Transcriptome in Mouse Early Embryos (DBTMEE)	Park et al. (2015)	http://dbtmee.hgc.jp
DAVID	Laboratory of Human Retrovirology and Immunoinformatics (LHRI)	https://david.ncifcrf.gov/
HOMER	Heinz et al. (2010)	http://homer.ucsd.edu/homer/
Ingenuity Pathway Analysis (IPA)	QIAGEN	N/A
Custom Source Code	Github Folder:s Miyamoto-lab/NT-ETR	https://github.com/Miyamoto-lab/NT-ETR; https://doi.org/10.5281/zenodo.8475

### Resource availability

#### Lead contact

Further information and requests for resources and reagents should be directed to and will be fulfilled by the Lead Contact, Kei Miyamoto (kmiyamo@waka.kindai.ac.jp).

#### Materials availability

All unique/stable reagents generated in this study are available from the Lead Contact with a completed Materials Transfer Agreement.

### Experimental model and subject details

#### Animals

Mice (C57BL/6 and DBA/2 strains) at 8–10 weeks of age were purchased from CLEA Japan (Tokyo, Japan) or Japan SLC (Shizuoka, Japan) and maintained in light-controlled, air-conditioned rooms. C57BL/6 female and DBA/2 male mice were used for *in vitro* fertilization. This study was carried out in strict accordance with the recommendations in the Guidelines of Kindai University for the Care and Use of Laboratory Animals. Experimental protocols were approved by the Committee on the Ethics of Animal experiments of Kindai University (Permit Number: KABT-31-003). All mice were sacrificed by cervical dislocation and all efforts were made to minimize suffering and to reduce the number of animals used in the present study.

Muscle tissues of a female *Oryx dammah* were collected from a carcass for necropsy at Adventure World Zoo (Wakayama, Japan), and frozen until use. The animal welfare and ethics were in accordance with guidelines adopted by the Japanese Association of Zoos and Aquariums.

#### Cell culture

A transgenic mouse ES cell line, termed NeoR5 (Miyamoto et al., 2011), harboring 20 to 30 copies of Lac operator repeats, the *Oct4* regulatory region and neomycin resistance gene was cultured without feeder cells in GMEM (078-05525, FUJIFILM Wako Pure Chemical Corporation, Osaka, Japan) containing recombinant mouse leukemia inhibitory factor (LIF2010, SIGMA-Aldrich), 2-mercaptoethanol, non-essential amino acids, and 20% KnockOut Serum Replacement (all from Thermo Fisher Scientific) to maintain the undifferentiated state. Differentiation of the ES cell line was induced by addition of 0.1 μM retinoic acid (R2625-50MG, SIGMA-Aldrich). After 4 days of culture, differentiated NeoR5 cells were trypsinized, collected and suspended in the ES cell culture medium until being subjected to NT-ETR.

The C2C12 cell line, an immortalized mouse myoblast cell line, derived from C3H strain mouse was cultured in DMEM (D5796, SIGMA-Aldrich) containing 10% fetal bovine serum (FBS) (172012, SIGMA-Aldrich) and antibiotics penicillin and streptomycin (100 μg/ml; Nacalai tesque, INC., Kyoto, Japan). Semi-confluent cells were trypsinized, collected and suspended in the growth medium until being subjected to NT-ETR.

Primary cells derived from muscles of post-mortem female *Oryx dammah* were cultured in DMEM containing 10% FBS and antibiotics penicillin, streptomycin (each 100 μg/ml) and 5 μg/ml fungizone (15290018, Thermo Fisher Scientific). The cultured *Oryx dammah* cells after three times of passages were trypsinized, collected and suspended in the growth medium without fungizone until being subjected to NT-ETR.

### Method details

#### *In vitro* fertilization (IVF) and embryo culture

Collection of spermatozoa, oocytes, and zygotes were performed as described in previous studies (Higuchi et al., 2019; Okuno et al., 2020). Briefly, spermatozoa were collected from the cauda epididymis of DBA/2 fertile male mice (>8 weeks of age). The sperm suspension was incubated in human tubal fluid (HTF) medium for 1.5 hours to allow for capacitation at 37°C under 5% CO_2_ in air. Oocytes were collected from the excised oviducts of C57BL/6 female mice (>8 weeks of age) that were superovulated with pregnant mare serum gonadotropin (PMSG; Serotropin, ASKA Pharmaceutical Co., Tokyo, Japan) and 48 hours later, human chorionic gonadotropin (hCG; ASKA Pharmaceutical Co.). Cumulus-oocyte complexes were recovered into pre-equilibrated HTF medium. The sperm suspension was added to the oocyte cultures and morphologically normal zygotes were collected 2 hours post insemination (hpi). The zygotes were cultured in potassium simplex optimized medium KSOM (ARK Resource, Kumamoto, Japan) at 37°C under 5% CO_2_ in air.

#### Preparing arrested recipient embryos for NT

To inhibit cell division of 4-cell embryos, they were cultured in KSOM medium containing 0.01, 0.1, 0.5, or 1.0 μg/ml demecolcine (D7385, SIGMA-Aldrich, St. Louis, SG) dissolved in ethanol from 40 hpi for 54 hours. Developmental progression of the embryos was checked in each concentration morphologically every 6 hours, and ratios of arrested 4-cell embryos were calculated. For inducing the cell cycle arrest of 2-cell embryos, 2-cell embryos were treated with 0.1 μg/ml demecolcine from 21 hpi onwards.

#### DNA replication assays with EdU incorporation

Four-cell embryos of BDF1 (C57BL/6 × DBA/2) mice were cultured in KSOM medium containing 0.1 μg/ml demecolcine and 10 mM EdU solution (Click-iT EdU Alexa Fluor 488 Imaging Assay; C10337, Thermo Fisher Scientific, Waltham, MA) from 40 to 43, 43 to 46, or 46 to 49 hpi. Cultured embryos were fixed in 4% paraformaldehyde (PFA)/PBS at room temperature for 15 min, and were washed three times by 0.01% polyvinyl alcohol (PVA)/PBS. Embryos were next treated with 0.5% triton X-100/PBS at room temperature for 20 min, followed by washing three times with 3% BSA/PBS. EdU was labeled within 100 μl drops of the Click-iT reaction cocktail (C10337, Thermo Fisher Scientific). The embryos were then washed with 3% BSA/PBS three times and mounted onto a slide under a coverslip in the VECTASHIELD Mounting Medium with DAPI (H-1200, Vector Laboratories, Inc., Burlingame, CA). EdU and DAPI signals were observed by confocal laser scanning microscopy (LSM800, Carl Zeiss, Germany) with ×400 magnification, equipped with a laser module (405/488/561/640 nm) and GaAsP detector. Photographs were taken at the same contrast, brightness, and exposure settings within the same experiments. Z-slice thickness was determined by using the optimal interval function in the ZEN software (Carl Zeiss). Using ZEN software, fluorescence intensities of both EdU and DAPI within each nucleus were determined, and relative values to the mean nuclear fluorescence intensity for EdU and DAPI of 46-49 h samples were calculated. The Tukey's honestly significant difference test (Tukey's HSD) was used to test statistically significant differences among the means of fluorescence intensities (P < 0.05).

#### NT-ETR

When NeoR5 cells were used as donor cells, 200 ng/μl LacR-mCherry mRNA solution was injected into the IVF-derived BDF1 embryos at 6 hpi. The mCherry signals within nuclei were confirmed by using fluorescence microscope at 8–10 hpi. The 4-cell embryos were cultured in KSOM medium containing 0.1 μg/ml demecolcine from 40 hpi. Of note, this demecolcine treatment results in developmental arrest of 4-cell embryos at G2/M phase and this arrest is also observed when the embryos are transferred to the demecolcine-containing medium from 43 hpi onwards. The incubated 4-cell embryos were moved to M2 medium (ARK Resource) containing 0.1 μg/ml demecolcine at 46 hpi just before NT. Inactivated hemagglutinating virus of Japan envelope (HVJ-E) solution (CF004, Ishihara Sangyo Kaisha, LTD., Japan) was aspirated into a glass capillary, and subsequently two donor NeoR5 cells were picked up with a minimal volume of cell suspension medium in order to avoid the dilution of HVJ-E solution. The glass capillary containing cells and HVJ-E solution was inserted into perivitelline space of recipient embryos via a small hole of zona pellucida opened by the XYClone laser system (271348, Hamilton Thorne, Beverly, MA). Then, two NeoR5 donor cells together with two to three times more volume of HVJ-E solution than donor cells were placed adjacent to one of blastomeres of recipient BDF1 4-cell embryos. The reconstructed embryo was left in M2 medium for 5 min. Finally, the NT embryos were further cultured in KSOM medium containing 0.1 μg/ml demecolcine for 2 and 24 hours and subjected to immunofluorescence staining as depicted in Figure 2A.

When C3H strain mouse-derived C2C12 cells or the *Oryx dammah* cells were used for NT-ETR, BDF1 4-cell embryos were cultured in KSOM medium containing 0.1 μg/ml demecolcine from 40 to 43 hpi till 46 hpi. NT methods follow the ones described for the NeoR5 donors. Briefly, two donor cells together with HVJ-E solution were injected and placed adjacent to one of blastomeres of recipient BDF1 4-cell embryos. The reconstructed embryo was left in M2 medium for 5 min. Subsequently, the NT embryos were further cultured in KSOM medium containing 0.1 μg/ml demecolcine with or without 2.5 μg/ml α-amanitin (A2263-1MG, MERCK, Darmstadt, Germany) for 2 and 24 hours.

For NT to 2-cell embryos, C2C12 cells were used. BDF1 2-cell embryos were cultured in KSOM medium containing 0.1 μg/ml demecolcine from 21 hpi till 45 hpi (early 2-cell NT) or from 21 hpi till 54 hpi (late 2-cell NT) as depicted in Figure 6A. NT methods follow the ones described for the NeoR5 donors. Briefly, two donor cells together with HVJ-E solution were injected and placed adjacent to one of blastomeres of recipient BDF1 2-cell embryos. The reconstructed embryo was left in M2 medium for 5 min. Subsequently, the NT embryos were further cultured in KSOM medium containing 0.1 μg/ml demecolcine with or without 2.5 μg/ml α-amanitin for 24 hours.

#### Live cell imaging

NT embryos pre-loaded with LacR-mCherry were observed by live cell imaging. After NT of NeoR5 cells, such embryos were cultured in KSOM medium containing 0.1 μg/ml demecolcine for 2 hours and subsequently transferred to drops of KSOM medium with demecolcine on a glass-bottom dish (MatTek). The embryos were then placed in an incubation chamber (Tokai Hit) at 37°C under 5% CO_2_, which was set on a confocal microscope (LSM800, Carl ZEISS). Fluorescence signals for mCherry were detected with 561 nm laser irradiation (z-stacks of 69 slices with an interval of 1 μm). Time series were performed with an interval of 10 min. Images were analyzed using the ZEN software (Carl ZEISS).

#### Immunofluorescence staining

NT embryos were fixed in 4% PFA/PBS at room temperature for 20 min, and were washed three times by 0.01% PVA/PBS. Samples were next treated with 0.5% triton X-100/PBS at room temperature for 20 min, followed by washing three times with 3% BSA/PBS. The samples were blocked in 3% BSA/PBS for 1 hour at room temperature, then incubated with primary antibodies (anti-H3K4me3 [39159, Active Motif, Carlsbad, CA; ab8580, Abcam, Cambridge, MA] diluted both in 1:500, anti-H3K9me3 [ab8898, Abcam, Cambridge, MA] diluted in 1:300, anti-H3S10ph [ab5176, Abcam] diluted in 1:200 or anti-RNA polymerase II phosphorylated at serine 2 [cloneH5: MMS-129R, Covance, Princeton, NJ] diluted in 1:100) at 4°C overnight. Following three times washes by 3% BSA/PBS, samples were further incubated in the dark with Alexa Fluor 488-labeled goat anti-rabbit IgG antibody (1:2,000; A11008, Thermo Fisher Scientific) or Alexa Fluor 488-labeled goat anti-mouse IgM antibody (1:2,000; A21042, Thermo Fisher Scientific) at room temperature for 1 hour. The samples were washed with 3% BSA/PBS three times and then mounted on slides using VECTASHIELD Mounting Medium containing DAPI. The fluorescence signals were observed using a LSM800 microscope, equipped with a laser module (405/488/561/640 nm) and GaAsP detector, using the same contrast, brightness, and exposure settings within the same experiments. Z-slice thickness was determined by using the optimal interval function in the ZEN software.

#### RNA-seq analysis

Timings for sampling are shown in each corresponding Figure. A pool of three NT embryos for Figures 3 and 4, four NT embryos for Figure 5 and five NT embryos for Figures 6 and 7 were treated with acid Tyrode, followed by three times washes with 0.1% BSA/PBS, and were moved into 1×Reaction buffer from SMART-seq v4 Ultra Low Input RNA Kit (24888N, Takara). SMART-seq library preparation was performed using SMART-seq v4 Ultra Low Input RNA Kit and Nextera DNA Sample Preparation Kit (FC-131-1024, illumine, San Diego, CA) according to the vendor’s instruction.

Paired-end sequencing (50 bp + 25 bp) was obtained by the NextSeq platform (Illumina). Raw reads were first subjected to filtering to remove low quality reads using Trimmomatic (Bolger et al., 2014). Reads of less than 20 bases and unpaired reads were also removed. Furthermore, adaptor, polyA, polyT and polyG sequences were removed using Trim Galore tool (https://www.bioinformatics.babraham.ac.uk/projects/trim_galore/) and Cutadapt (Martin, 2011). The remaining reads were mapped to the mouse mm10 or *Oryx dammah* genome (Humble et al., 2020) by the STAR tool (Dobin et al., 2013). Gene expression values were calculated as fragments per kilobase of exon per million mapped fragments (FPKM). FPKM values for triplicates in each condition were subjected to statistical analyses to extract differentially expressed genes (DEGs, fulfilling two criteria: four-fold change cutoff (α-amanitin (-) vs. α-amanitin (+)) and the padj < 0.05 in the Benjamini-Hochberg procedure for the FPKM values of these two groups) using DESeq2 (Love et al., 2014) by normalizing with ERCC RNA Spike-In (4456740, Thermo Fisher Scientific) counts.

For assignment of transcripts to individual mouse strains (C57BL/6, DBA/2 or C3H), trimmed reads were aligned to a merged cDNA fasta file of the three strains (individual files available from Sanger site https://www.sanger.ac.uk/data/mouse-genomes-project/) using Kallisto (https://github.com/pachterlab/kallisto/releases) with 100 bootstrapped samples (Bray et al., 2016). Transcript counts were then separated by strain and differential expression quantified using sleuth (Pimentel et al., 2017). Transcripts were compared with transcripts of each developmental stage listed in the Database of Transcriptome in Mouse Early Embryos (DBTMEE) (Park et al., 2015). The genes transcribed in each strain specifically were subjected to gene ontology (GO) classification using the DAVID gene analysis tool (https://david.ncifcrf.gov). Furthermore, transcription factor binding site motifs enriched among the strain (C57BL/6, DBA/2 or C3H)-specific expressed genes’ TSSs were identified using the findMotifsGenome.pl in HOMER (Heinz et al., 2010). Each gene list was further subjected to an Ingenuity Pathway Analysis (IPA; QIAGEN, Redwood City, CA). Using IPA, enriched canonical pathways, upstream transcriptional regulators, and diseases and biological functions were investigated. In order to compare transcriptomes of injected C2C12 nuclei with donor C2C12 cells, RNA-seq dataset of C2C12 cells (GEO: GSE49321; GEO: GSM1197387-GSM1197392) were used.

For mapping of reads following reprogramming of *Oryx dammah* donor cells using mouse embryos, trimmed reads were first aligned to the mouse genome mm10 using STAR aligner (Dobin et al., 2013). Unmapped reads were retained using the option --outReadsUnmappedFastx and these unmapped reads were aligned to the *Oryx dammah* genome (Humble et al., 2020) using STAR. Gene annotation files (.gtf) for *Oryx dammah* were generated from gff3 files (Humble et al., 2020) using gffread function, and gene expression values were quantified (using --quantMode GeneCounts). Overlaps between *Oryx dammah* genes and regions of homology to human or bovine genes identified in the gff3 file were identified using bedtools intersect. Gene counts in triplicate were used to identify differentially expressed genes (DEGs, fulfilling two criteria: four-fold change cutoff (α-amanitin (-) vs. α-amanitin (+)) and the padj < 0.05 in the Benjamini-Hochberg procedure for the count values of these two groups) using DESeq2 (Love et al., 2014).

#### RNA extraction and RT-PCR

RNA of three NT embryos was extracted using Arcturus PicoPure RNA isolaction Kit (108080044, Thermo Fisher Scientific) as described by the manufacturer. Isolated RNAs were reverse-transcribed to cDNA using SuperScript Reverse Transcriptase (18080044, Thermo Fisher Scientific) with random hexamers (N8080127, Thermo Fisher Scientific). The PCR was carried out with *Lzic2*-specific primers (5′-tcgatgcggatgaatatgaa-3′ and 5′-gctagccttgtccgaagttg-3′), *Ktil2*-specific primers (5′-acaccaaaatgcatcccaat-3′ and 5′-gtttcccagggacagaacaa-3′), *Tmem144*-specific primers (5′-gttgccttggtggtcaactt-3′ and 5′-ggcttggacacttcttctgc-3′), *Rnd3*-specific primers (5′-cctgtgggacacttcaggtt-3′ and 5′-tcttcgctttgtcctttcgt-3′), *Plk2*-specific primers (5′-agccagcggaaagatacaga-3′ and 5′-taaacagcaagggagcaacc-3′), *Aqp3*-specific primers (5′-accatcaacttggcttttgg-3′ and aacgatggccagtacacaca-3′) or *Actb*-specific primers (5′-tccttcttgggtatggaatcctgt-3′ and 5′-tggcatagaggtctttacgga-3′). Each PCR reaction was performed under the following conditions: for *Lzic, Ktil2, Tmem144, Rnd3, Plk2* and *Aqp3*, 98°C 30 sec; 35 cycles of 98°C, 10 s; 59°C, 30 s; 72°C, 30 sec; final extension 72°C, 10 min; for *Actb*, 98°C 30 sec; 35 cycles of 98°C, 10 s; 64°C, 30 s; 72°C, 30 sec; final extension 72°C, 10 min.

#### siRNA knockdown of Zfp281

For investigating a function of ZFP281, specific siRNA against *Zfp281* (4390771[s105405], Thermo Fisher Scientific) was injected to IVF embryos at 6 hpi, and the injected embryos were used for NT-ETR as described above. Pre-designed negative control siRNA (RNAi Inc.) was used as a control. The NT embryos were further cultured in KSOM medium containing 0.1 μg/ml demecolcine for 24 hours, and four NT embryos were subjected to quantitative RT-PCR (qRT-PCR) using following primers: *Kti12*-specific primers (5′-agtctcagcctctagcctctgg-3′ and 5′-tggcctgatccaactggtg-3′), *Rnd3*-specific primers (5′-aagcggaacaaatcgcagag-3′ and 5′-gctaggcatgtgcgaaatcc-3′), *Plk2*-specific primers (5′-ctgtgtaatgtatacgatgctgctagg-3′ and 5′-tttgtggtttcgaatggaggt-3′), *Aqp3*-specific primers (5′-gctgtgaccttcgcaatgtg-3′ and 5′-cagcttgatccagggctctc-3′), *Zfp281*-specific primers (5′-cgttaccagacgtagttgggc-3′ and 5′-atgccaagtgagccacctg-3′) and 28S rRNA-specific primer (5′-aaggctaaataccggcacga-3′ and 5′-tcttaacggtttcacgccct-3′). PCR reactions were performed under the following condition using 7300 Real Time System (Thermo Fisher Scientific): 95°C 30 s; 40 cycles of 95°C, 5 s; 60°C, 31 s with the dissociation process. Two-tailed Student’s t-test was used to assess statistical significances. A P value < 0.05 was considered as statistically significant.

### Quantification and statistical analysis

All of the statistical methods for each experiment can be found in the Figure legends as well as in the Method Details section. For Figure 1D, the Tukey's honestly significant difference test (Tukey's HSD) was used to assess statistically significant differences among the means of fluorescence intensities. For Figure 4G, Student’s t-test was used to evaluate statistical significances of gene expression differences. For Figure 6B, Fisher’s exact test was performed to assess statistically significant differences of arrested embryos between demecolcine-treated and non-treated samples.

## Data Availability

• RNA-seq data have been deposited at GEO and are publicly available as of the date of publication. This paper also analyzes existing, publicly available data. These accession numbers for the datasets are listed in the key resources table. Microscopy data reported in this paper will be shared by the lead contact upon request. • All original code has been deposited at GitHub and is publicly available as of the date of publication. DOIs are listed in the key resources table. • Any additional information required to reanalyze the data reported in this paper is available from the lead contact upon request.

## Supplemental information

Document S1. Figures S1–S6

Table S1. Differentially expressed genes (DEGs) between NT embryos with α-amanitin and those without α-amanitin, related to Figure 3

Table S2. Top 30 upstream regulators identified by Ingenuity Pathway Analysis (IPA) and their target molecules in dataset, related to Figure 3

Table S3. Differentially expressed repeat sequences between NT embryos with α-amanitin and those without α-amanitin, related to Figure 3

Table S4. B6-specific DEGs, related to Figure 4

Table S5. C3H-specific DEGs, related to Figure 4

Table S6. DBA-specific DEGs, related to Figure 4

Table S7. Top 40 upstream regulators identified by IPA of B6-specific DEGs and their target molecules in dataset, related to Figure 4

Table S8. Top 40 upstream regulators identified by IPA of C3H-specific DEGs and their target molecules in dataset, related to Figure 4

Table S9. Top 40 upstream regulators identified by IPA of DBA-specific DEGs and their target molecules in dataset, related to Figure 4

Table S10. Top 50 upstream regulators identified by IPA of differentially expressed *Oryx dammah* genes, related to Figure 7
