# Supplementary material for: Cell division- and DNA replication-free reprogramming of somatic nuclei for embryonic transcription
Source: iScience. 2021 Nov 3;24(11):103290. doi: 10.1016/j.isci.2021.103290 (PMC8609233; doi:10.1016/j.isci.2021.103290)
Supplement: Document S1. Figures S1–S6 [file mmc1.pdf]

iScience, Volume *24*

Supplemental information

Cell division- and DNA replication-free reprogramming of somatic nuclei for embryonic transcription

Junko Tomikawa, Christopher A. Penfold, Takuma Kamiya, Risa Hibino, Ayumi Kosaka, Masayuki Anzai, Kazuya Matsumoto, and Kei Miyamoto

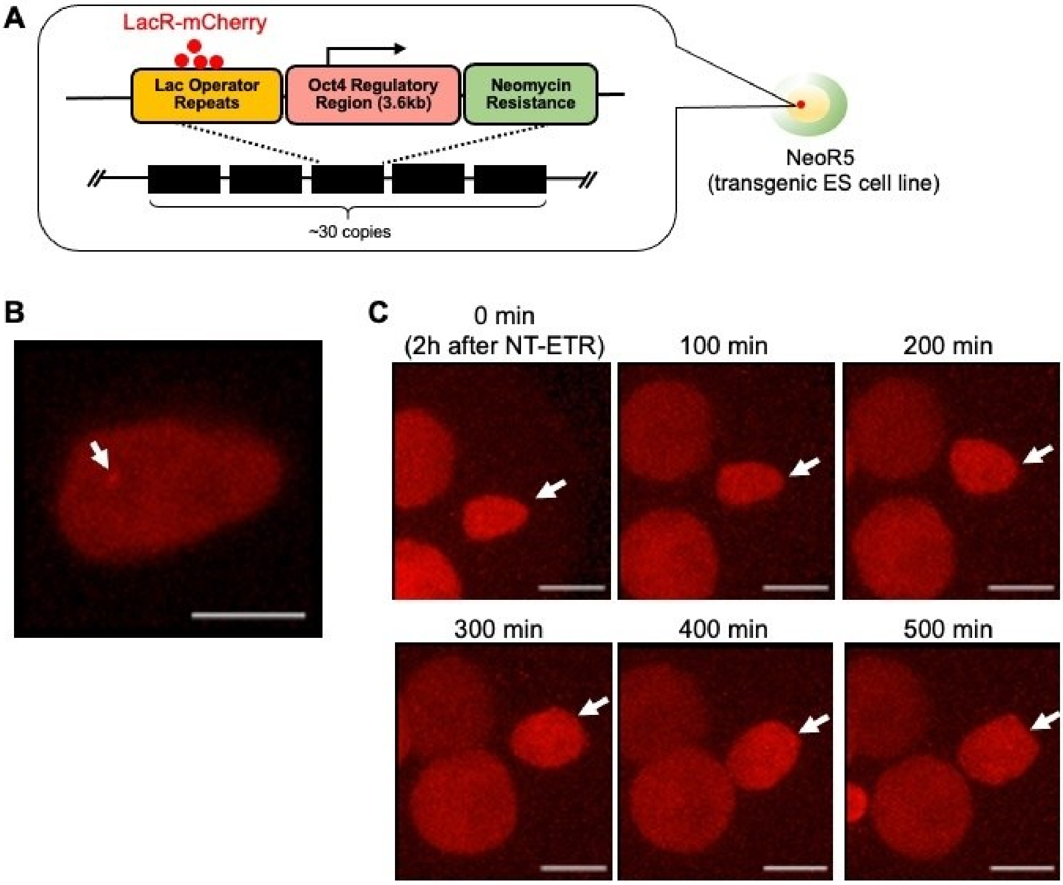

Figure S1: Gradual nuclear swelling of a differentiated cell nucleus injected into a 4-cell embryo, related to Figure 2. (A) A diagram shows a transgenic ES cell line, termed NeoR5, that harbors 20 to 30 copies of Lac operator repeats, the *Oct4* gene regulatory region and neomycin resistance gene. (B) Accumulation of mCherry-LacR (arrow) marks the injected NeoR5 cell nucleus, carrying lac operator repeats as depicted in Figure S1A. Scale bar, 5 μm. (C) Live cell imaging traces the swelling process of a differentiated cell nucleus injected into a 4-cell embryo pre-loaded with mCherry-LacR. Arrows indicate the injected nucleus. Times after NT-ETR are shown. Scale bars, 10 μm. Data presented are the representative of two independent experiments.

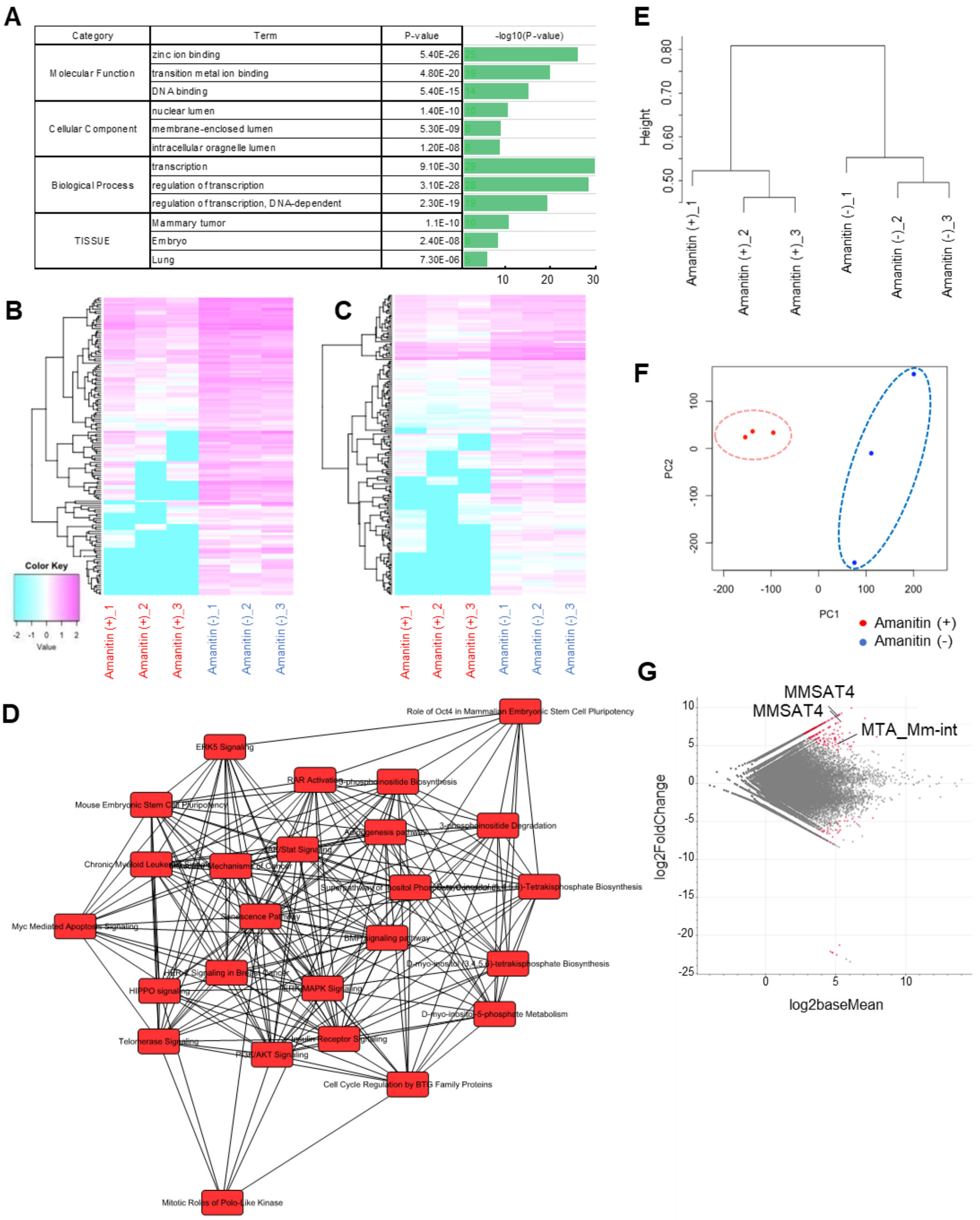

Figure S2: Embryonic gene programs are activated after NT-ETR, related to Figure 3. (A) Enriched terms of GO analyses using DEG lists obtained from RNA-seq of the reconstructed embryos after NT-ETR. (B) Heatmap shows the expression levels of overlapped genes between DEGs and 2-cell transient genes (138 genes in Figure 3E). (C) Heatmap shows the expression levels of overlapped genes between DEGs and 4-cell transient genes (169 genes in Figure 3E). (D) Canonical pathways predicted by Ingenuity Pathway Analysis (IPA) using DEGs between α-amanitin-treated and non-treated NT embryos. (E) Hierarchical clustering dendrogram generated using the expression profiles of repeat elements in NT embryos cultured with or without α-amanitin. (F) PCA of repeat element expression profiles. Red and blue dots indicate the groups cultured with or without α-amanitin, respectively. (G) An MA plot on the repeat element expression levels. Red dots are the DEGs fulfilling two criteria: four-fold change cutoff (α-amanitin (-) vs. α-amanitin (+)) and the padj < 0.05. Some selected repeat elements are shown on the plot. Three independent biological replicates were performed.

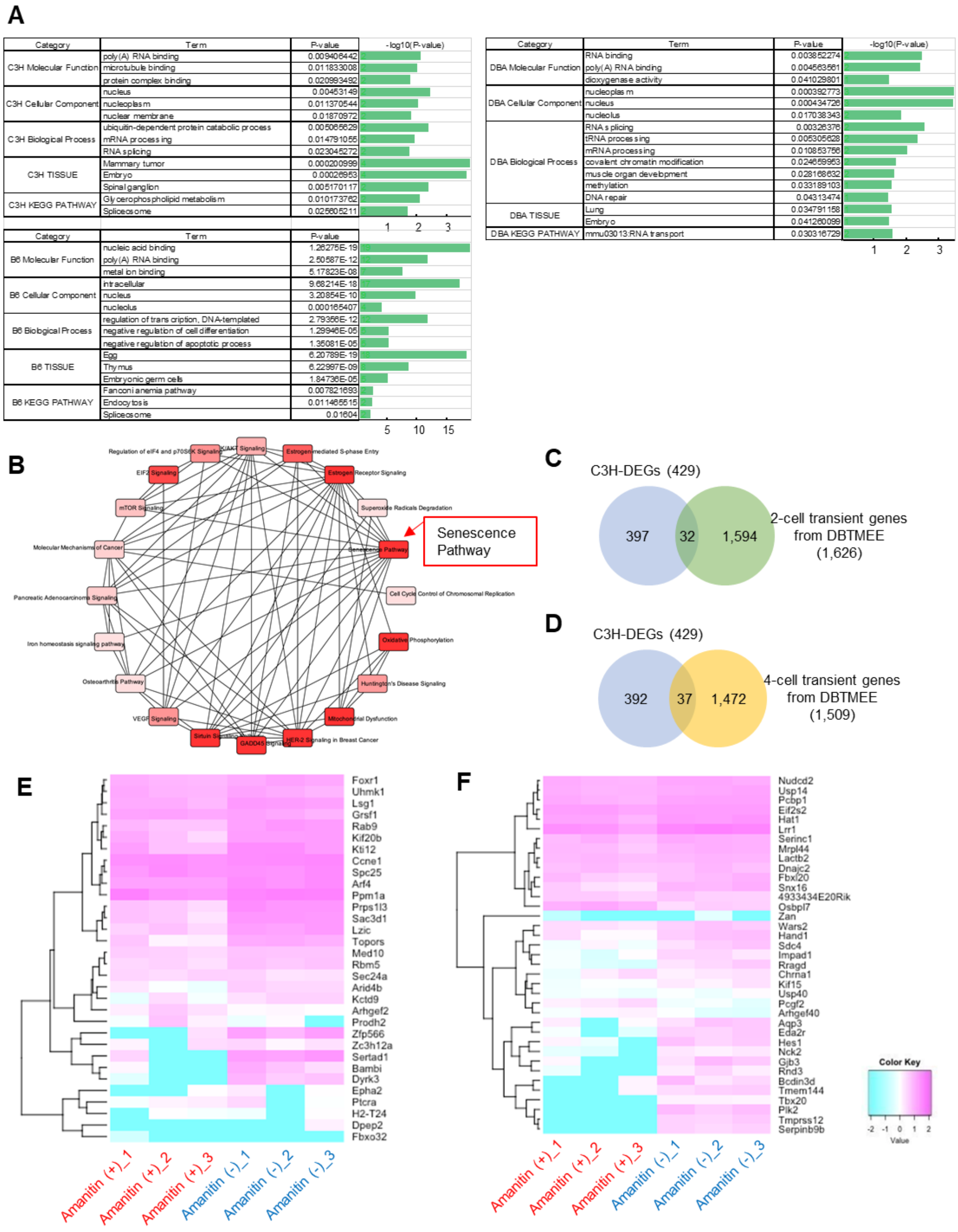

Figure S3: Reprogrammed transcripts from genomes of different mouse strains after NT-ETR, related to Figure 4. (A) Enriched GO terms in transcripts from the genome of each strain. IVF-derived BDF1 mouse 4-cell embryos were fused with C2C12 cells derived from C3H strain mouse. All transcripts from the NT embryos were sorted into each strain (C57BL/6 [B6], DBA/2 or C3H), and DEGs of each strain were detected as shown in Figure 4A. Using these gene lists, enriched GO terms were found based on a value of $p < 0.05$. (B) Canonical pathways predicted IPA using downregulated genes after NT-ETR in Figure 4B (cluster 1). (C and D) Venn diagrams showing the numbers of total and overlapping genes between the C3H DEGs and 2- or 4-cell transient genes. (E and F) Heatmap of overlapped genes between C3H DEGs and 2- (32 genes) or 4-cell transient genes (37 genes). Most of the 2-cell and 4-cell transient genes were transcriptionally activated after NT-ETR. Three independent biological replicates were performed.

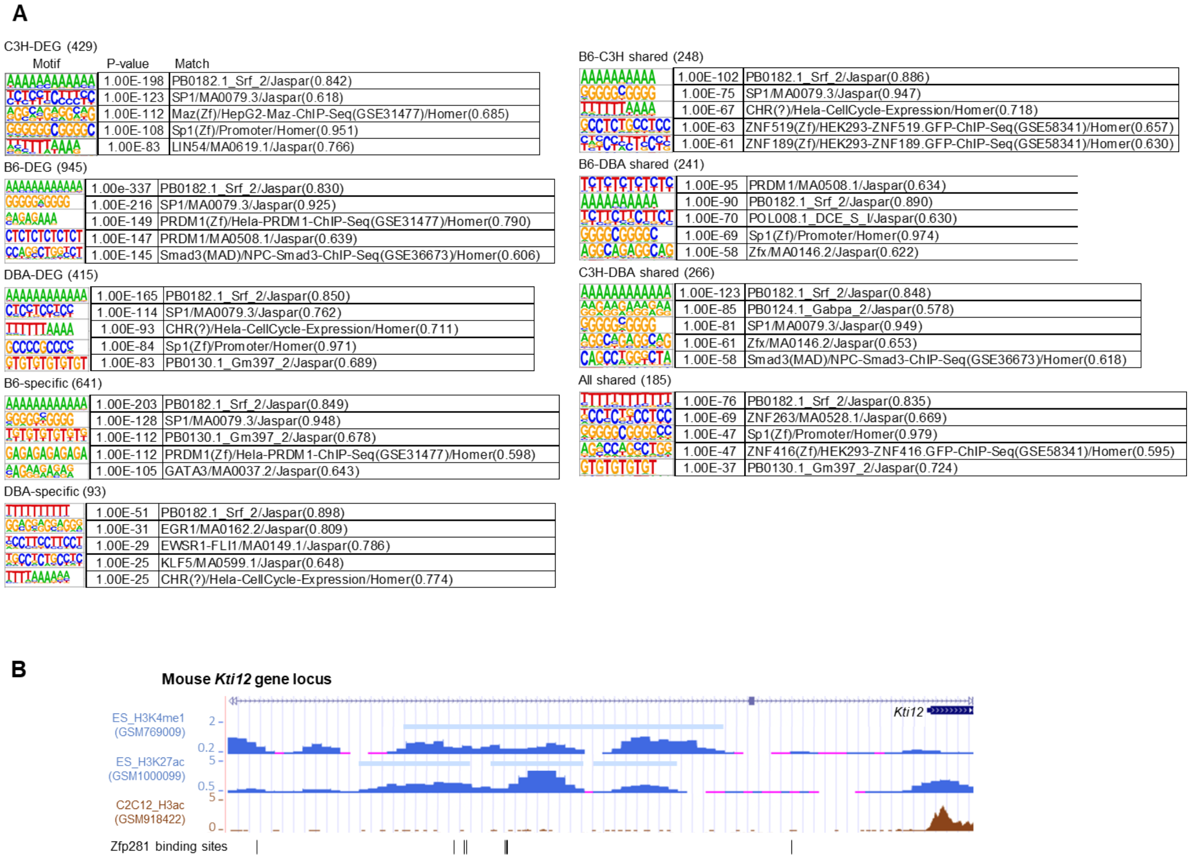

C3H-DEG (429)

P-value	Match
1.00E-198	PB0182.1_Srf_2/Jaspar(0.842)
1.00E-123	SP1/MA0079.3/Jaspar(0.618)
1.00E-112	Maz(Zf)/HepG2-Maz-ChIP-Seq(GSE31477)/Homer(0.685)
1.00E-108	Sp1(Zf)/Promoter/Homer(0.951)
1.00E-83	LIN54/MA0619.1/Jaspar(0.766)

B6-DEG (945)

P-value	Match
1.00e-337	PB0182.1_Srf_2/Jaspar(0.830)
1.00E-216	SP1/MA0079.3/Jaspar(0.925)
1.00E-149	PRDM1(Zf)/Hela-PRDM1-ChIP-Seq(GSE31477)/Homer(0.790)
1.00E-147	PRDM1/MA0508.1/Jaspar(0.639)
1.00E-145	Smad3(MAD)/NPC-Smad3-ChIP-Seq(GSE36673)/Homer(0.606)

DBA-DEG (415)

P-value	Match
1.00E-165	PB0182.1_Srf_2/Jaspar(0.850)
1.00E-114	SP1/MA0079.3/Jaspar(0.762)
1.00E-93	CHR(?)/Hela-CellCycle-Expression/Homer(0.711)
1.00E-84	Sp1(Zf)/Promoter/Homer(0.971)
1.00E-83	PB0130.1_Gm397_2/Jaspar(0.689)

B6-specific (641)

P-value	Match
1.00E-203	PB0182.1_Srf_2/Jaspar(0.849)
1.00E-128	SP1/MA0079.3/Jaspar(0.948)
1.00E-112	PB0130.1_Gm397_2/Jaspar(0.678)
1.00E-112	PRDM1(Zf)/Hela-PRDM1-ChIP-Seq(GSE31477)/Homer(0.598)
1.00E-105	GATA3/MA0037.2/Jaspar(0.643)

DBA-specific (93)

P-value	Match
1.00E-51	PB0182.1_Srf_2/Jaspar(0.898)
1.00E-31	EGR1/MA0162.2/Jaspar(0.809)
1.00E-29	EWSR1-FLI1/MA0149.1/Jaspar(0.786)
1.00E-25	KLF5/MA0599.1/Jaspar(0.648)
1.00E-25	CHR(?)/Hela-CellCycle-Expression/Homer(0.774)

B6-C3H shared (248)

P-value	Match
1.00E-102	PB0182.1_Srf_2/Jaspar(0.886)
1.00E-75	SP1/MA0079.3/Jaspar(0.947)
1.00E-67	CHR(?)/Hela-CellCycle-Expression/Homer(0.718)
1.00E-63	ZNF519(Zf)/HEK293-ZNF519.GFP-ChIP-Seq(GSE58341)/Homer(0.657)
1.00E-61	ZNF189(Zf)/HEK293-ZNF189.GFP-ChIP-Seq(GSE58341)/Homer(0.630)

B6-DBA shared (241)

P-value	Match
1.00E-95	PRDM1/MA0508.1/Jaspar(0.634)
1.00E-90	PB0182.1_Srf_2/Jaspar(0.890)
1.00E-70	POL008.1_DCE_S_I/Jaspar(0.630)
1.00E-69	Sp1(Zf)/Promoter/Homer(0.974)
1.00E-58	Zfx/MA0146.2/Jaspar(0.622)

C3H-DBA shared (266)

P-value	Match
1.00E-123	PB0182.1_Srf_2/Jaspar(0.848)
1.00E-85	PB0124.1_Gabpa_2/Jaspar(0.578)
1.00E-81	SP1/MA0079.3/Jaspar(0.949)
1.00E-61	Zfx/MA0146.2/Jaspar(0.653)
1.00E-58	Smad3(MAD)/NPC-Smad3-ChIP-Seq(GSE36673)/Homer(0.618)

All shared (185)

P-value	Match
1.00E-76	PB0182.1_Srf_2/Jaspar(0.835)
1.00E-69	ZNF263/MA0528.1/Jaspar(0.669)
1.00E-47	Sp1(Zf)/Promoter/Homer(0.979)
1.00E-47	ZNF416(Zf)/HEK293-ZNF416.GFP-ChIP-Seq(GSE58341)/Homer(0.595)
1.00E-37	PB0130.1_Gm397_2/Jaspar(0.724)

Figure S4: TFs potentially involved in transcriptional reprogramming after NT-ETR, related to Figure 4. (A) Enriched sequence motifs identified by the HOMER de novo motif analyses using the DEG lists in each strain of genome after NT-ETR. The top five de novo motifs are shown. The number of genes in each category is shown in parenthesis and the number corresponds to the ones described in Figure 4A. (B) Track view of ChIP-seq (H3K4me1, H3K27ac and H3ac) from datasets of GSM769009, GSM1000099 and GSM918422, and predicted ZFP281 binding sites at the *Kti12* upstream region in ES cells and C2C12 cells.

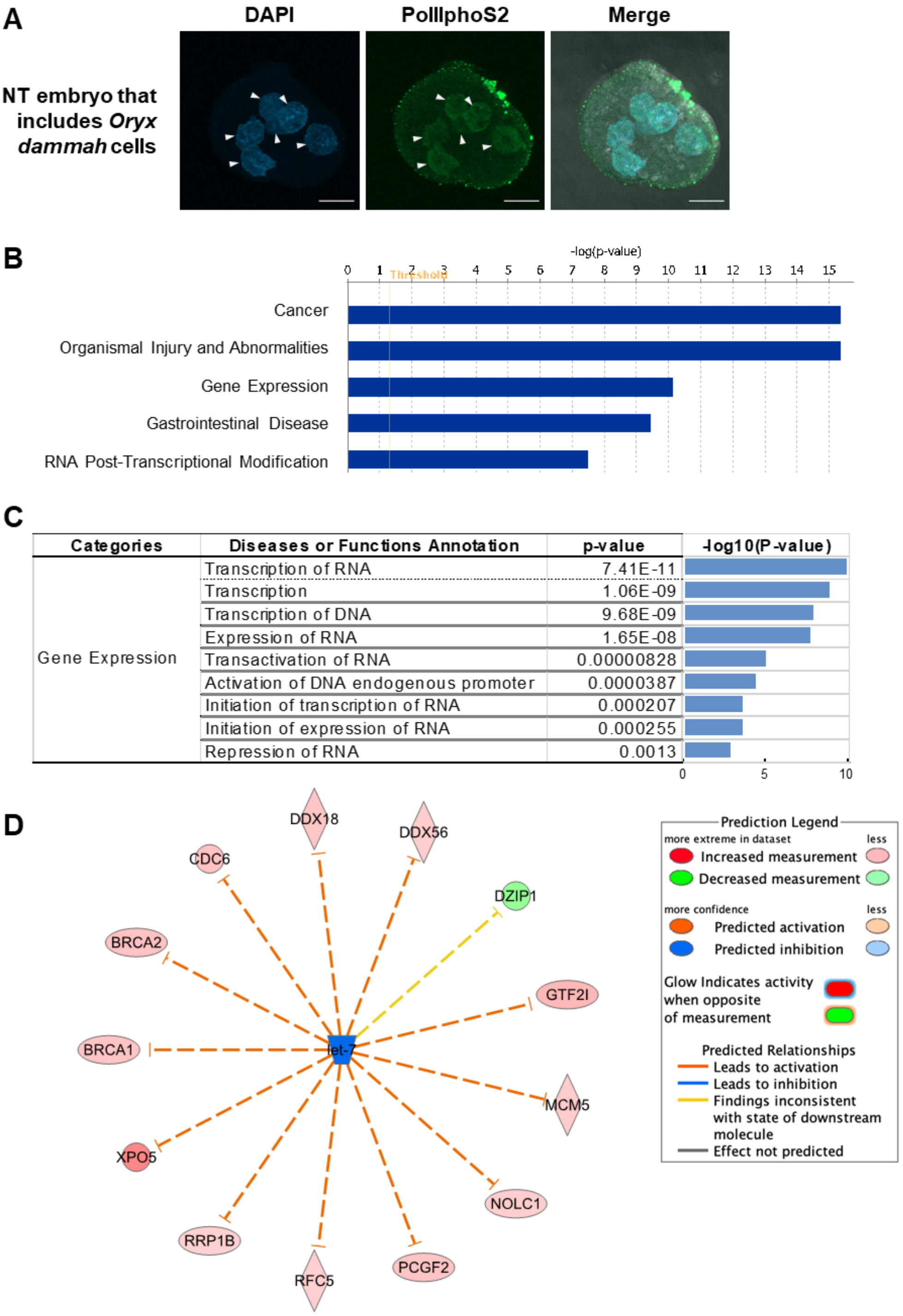

Figure S5: Reprogramming of a transcriptional state of *Oryx dammah* cells, related to Figure 6. (A) Immunostaining of PolIIphoS2 in a NT embryo in which two *Oryx dammah* cells were injected into a mouse 4-cell stage embryo. PolIIphoS2 signals were detected in all nuclei that included those from *Oryx dammah* cells, and all nuclei are marked by arrowheads. DNA was stained with DAPI (blue). Three independent experiments were performed. Scale bars indicate 20 µm. (B) Top 5 diseases and biological functions enriched by IPA for the 729 upregulated *Oryx dammah* DEGs between NT embryos with α-amanitin and those without α-amanitin. (C) Functional annotation of the enriched term "Gene expression" in Figure S5B. (D) Downstream effects analysis of let-7 miRNA revealed by IPA. Gene regulatory networks of let-7 in NT embryos are predicted.

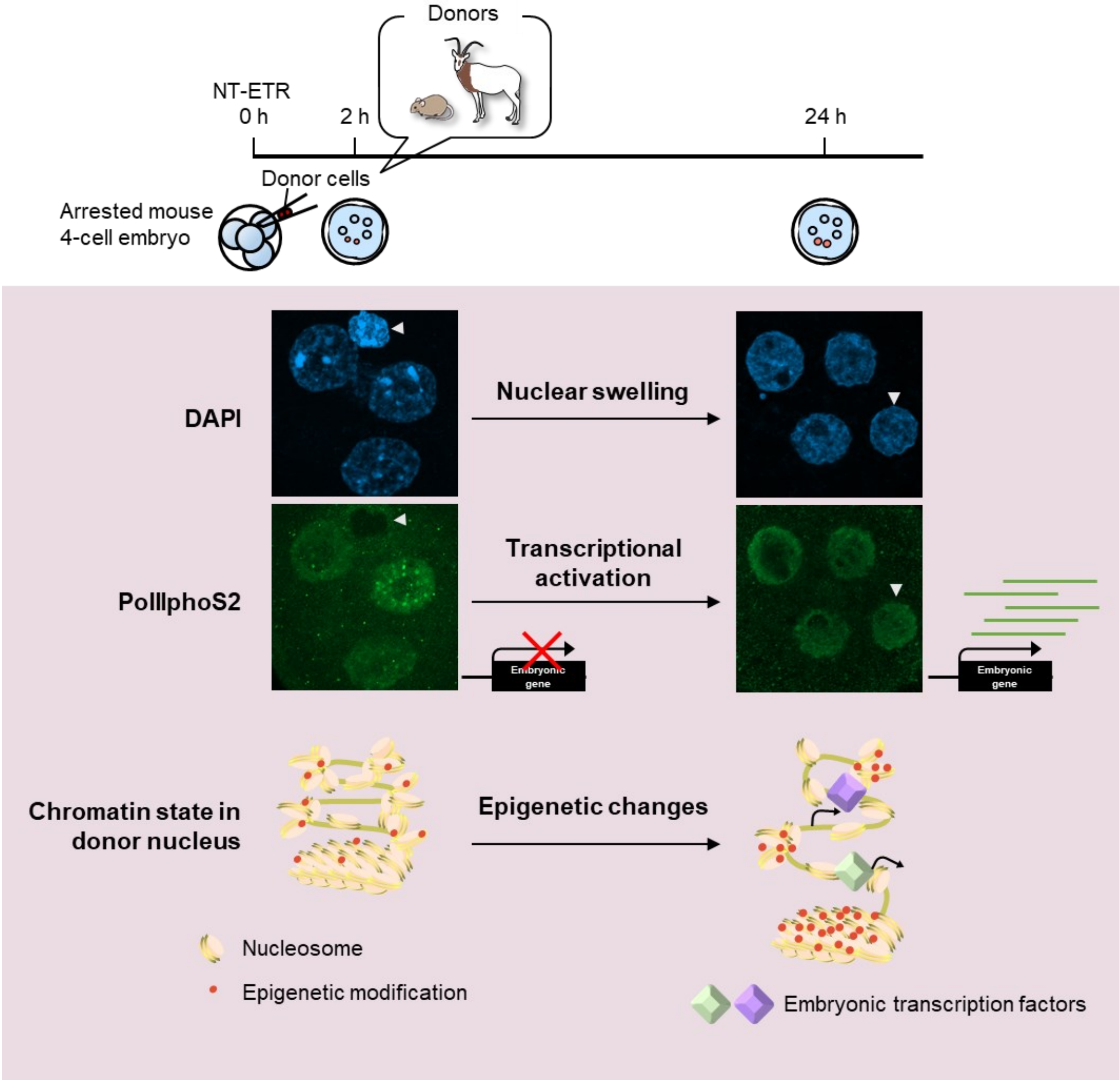

Figure S6: Mouse embryos arrested at the 4-cell stage elicit cross-species transcriptional reprogramming within a day without cell division and DNA replication, related to Figures 1-7. The model for cell division and replication-free reprogramming is depicted. By using NT-ETR, we have successfully induced transcriptional activation of embryonic genes from the intraspecies and cross-species genomes within a day. In the donor cell nucleus, chromatin structures are dynamically reprogrammed in parallel with nuclear swelling. Embryonic transcription factors such as ZFP281 are responsible for this cell division- and replication-free reprogramming. Arrowheads in the immunofluorescent images indicate the injected donor cell nucleus, which becomes transcriptionally active after NT. The immunofluorescent images are also used in Figure 2C.
